# Phenotypic and genotypic characterization of antibiotic-resistant bacteria from Swiss ready-to-eat meat products

**DOI:** 10.3389/fmicb.2025.1649307

**Published:** 2025-09-10

**Authors:** Lisa Thoenen, Joerg Hummerjohann, Livia Schwendimann, Elisabet Marti

**Affiliations:** ^1^Food Microbial Systems, Agroscope, Bern, Switzerland; ^2^Swiss Quality Testing Services (SQTS), Dietikon, Switzerland

**Keywords:** antimicrobial resistance (AMR), antibiotic resistance genes (ARGs), ready-to-eat meat products, whole-genome sequencing, antibiotic susceptibility testing

## Abstract

Antimicrobial resistance is a global health concern, which is partly driven by rising meat consumption, which has led to the intensive farming of livestock that relies on antibiotics. ready-to-eat animal products can carry antibiotic-resistant bacteria, posing risks to humans since they are often consumed without further cooking. While countries such as Switzerland limit antibiotic use in agriculture, contamination of meat with antibiotic-resistant bacteria can still occur during meat processing, and non-antibiotic agents such as heavy metals may contribute to the co-selection of resistance. This study aimed to characterize antibiotic-resistant bacteria in ready-to-eat meat products from various Swiss butcheries. Presumptive resistant bacteria were isolated using selective plating and analyzed phenotypically and genotypically. A total of 53 bacteria-antibiotic resistance combinations were identified, including Enterobacterales resistant to third-generation cephalosporins, vancomycin-resistant Enterococci, and one strain of methicillin-resistant *Staphylococcus aureus*. Of the 804 products sampled, 177 antibiotic-resistant bacteria were isolated, 148 of which showed multidrug resistance. Notably, these strains remained susceptible to last-resort antibiotics such as carbapenems and colistin. Whole-genome sequencing of 31 selected isolates revealed 164 antibiotic resistance genes spanning 25 classes, confirming resistance to beta-lactams, cephalosporins, and tetracyclines. We also detected genes conferring resistance to metals, suggesting co-selection pressures. Long-read sequencing revealed that the majority of the antibiotic resistance genes were chromosomal, while others were plasmid-encoded, indicating the potential for horizontal gene transfer. This study demonstrates that ready-to-eat meat products are reservoirs of antibiotic and metal resistance genes, as well as antibiotic-resistant bacteria, even at low levels. From a One Health perspective, our results highlight the importance of extending AMR surveillance across the food chain and underscore the need to include non-traditional bacterial indicators.

## 1 Introduction

The growing global demand for meat has led to increased reliance on intensive animal farming systems, which depend on antibiotics to maintain animal health. Globally, 73% of all antimicrobials sold are used in raising food-producing animals (Van Boeckel et al., 2017). This excessive use is considered a major driver of the emergence and spread of Antimicrobial resistance (AMR) (Kirchhelle, 2018), which is one of the leading public health concerns, accounting for 1.27 million deaths worldwide in 2019 (Murray et al., 2022). The use of antibiotics varies considerably between countries. In Switzerland, a total of 24 tons of antibiotics were used to treat animals in 2023, representing approximately 79% of total antibiotic consumption. Due to increased systematic recording of antibiotic use and resistance monitoring, overall antibiotic use has been steadily declining in recent years (Federal Office of Public Health and Federal Food Safety and Veterinary Office, 2024). Over the past 12 years, sales of antibiotics for livestock in Switzerland have decreased by 50% (Bundesamt für Lebensmittelsicherheit und Veterinärwesen BLV, 2024; Dattani et al., 2024). In livestock, the majority of antibiotics were prescribed to cattle (78.8%), followed by pigs (13.5%), small ruminants (1.1%), and poultry (0.8%). The most commonly used antibiotics in livestock are penicillins, followed by sulfonamides, and tetracyclines. Critically important antimicrobials for human medicine, including macrolides, fluoroquinolones, and third—and fourth—generation cephalosporins, are not allowed for use in livestock. Colistin sales are also reducing, which is significant since it has been used in recent years as a last-resort antibiotic against carbapenem-resistant Enterobacteriaceae (Federal Office of Public Health and Federal Food Safety and Veterinary Office, 2024). Given the widespread and varied use of antibiotics in food-producing animals, it is important to understand how this contributes to the spread of AMR.

According to the One Health approach, which recognizes the interconnectedness of human, animal, and environmental health, it is key to consider food as a means by which AMR can spread from animals and the environment to humans (Despotovic et al., 2023). Antibiotic-resistant bacteria (ARB) from livestock can be transmitted to humans either through direct contact (Trinh et al., 2018) or by consuming contaminated food such as meat, eggs, and dairy products (Rolain, 2013; Forslund et al., 2014; Rodrigues et al., 2020). A recent risk assessment in Switzerland identified raw meat as posing the highest risk of carrying antibiotic-resistant bacteria (Jans et al., 2018). Contamination of meat with pathogens and ARB can occur at various stages. During slaughter, for example, contamination may originate from the animal's hide or gastrointestinal tract (Weinroth et al., 2022). Commensal bacteria in meat can act as opportunistic pathogens or serve as reservoirs of antibiotic resistance genes (ARGs), carrying these genes on plasmids that can be transferred to other bacteria (Rolain, 2013). In Switzerland, the highest-risk combination was found to be the contamination of poultry meat with *E. coli* producing either extended-spectrum beta-lactamases (ESBL) or AmpC, as these enzymes confer resistance to beta-lactam antibiotics (Collineau et al., 2018). Furthermore, the same assessment raised concerns about *Enterococcus* resistant to tetracyclines or macrolides, as well as *Campylobacter jejuni* resistant to fluoroquinolones or macrolides (Collineau et al., 2018). More recently, a study detected ESBL-producing Enterobacterales in 10% of Swiss chicken meat (Gómez-Sanz et al., 2023). These findings are consistent with the observed decrease in antibiotic-resistant *E. coli* in poultry meat between 2014 and 2020, with a decline from 65.5% to 10.2% (Federal Office of Public Health and Federal Food Safety and Veterinary Office, 2022).

Unlike raw meat, which is usually cooked before consumption, ready-to-eat (RTE) meat products undergo processing methods such as curing, drying, or aging. These steps aim to inhibit or slow down the growth of harmful bacteria and spoilage microbes in the meat, allowing these products to be consumed without further cooking (Toldrá, 2017). However, bacteria present in the processing environment can contaminate RTE products during processing and ripening (Cobo-Díaz et al., 2021; Barcenilla et al., 2024). Processed meats, such as dry-aged, cured, and fermented sausages, harbor higher levels of ARGs, including those conferring resistance to beta-lactams, tetracyclines, and aminoglycosides, than raw meats (Cobo-Díaz et al., 2021; Barcenilla et al., 2024). Similarly, cutting can lead to an increase in ESBL-producing Enterobacterales and vancomycin-resistant *Enterococci* in pork meat (Cobo-Díaz et al., 2021). Certain persistent microbes are commonly found in the meat processing environment, which persist due to their ability to form biofilms and survive disinfection procedures (Zwirzitz et al., 2020). Therefore, RTE meat products, which are intended to be consumed uncooked, pose a higher risk of carrying and transmitting ARB from animals to humans than raw meat, which is usually cooked before consumption. This was identified as a knowledge gap in previous risk assessments (Jans et al., 2018).

Over time, bacteria have evolved various antibiotic resistance mechanisms as a survival strategy against the selective pressure exerted by antibiotics. Some of these mechanisms are “intrinsic,” whereby the cell uses genes it already possesses to survive antibiotic exposure. Bacteria can also acquire new mechanisms by *de novo* mutations or by horizontal gene transfer (HGT) of ARGs between bacteria of the same or different species (Darby et al., 2022; Murray et al., 2024). In addition to antibiotics, bacteria can also harbor resistance genes against biocides or metals (biocide and metal resistance genes, BMRGs). These non-antibiotic agents can select for antibiotic resistance through co-resistance, cross-resistance, or co-regulation (Murray et al., 2024). Co-resistance occurs when resistance genes are physically linked, for example, when ARGs and BMRG are located on the same plasmid. Cross-resistance is based on a mechanism that provides resistance to more than one agent, For example, multidrug efflux pumps. Co-regulation involves resistance genes that share a promoter or a regulator, for example, two-component systems responding to heavy metals and leading to antibiotic resistance (Dieppois et al., 2012; Gillieatt and Coleman, 2024; Murray et al., 2024). The co-selection of antibiotic resistance through environmental contaminants increases the spread and the persistence of AMR in the environment (Murray et al., 2024).

The spread of ARB in the environment has traditionally been monitored using culture-based isolation and phenotypic susceptibility testing. Whole-genome sequencing of resistant isolates has been employed to investigate the genetic elements driving the resistance phenotype and co-selection mechanisms in more detail (Baker et al., 2018; Djordjevic et al., 2023). Initially, genome sequencing yielded short reads (~150 bp), providing low resolution of specific genomic regions. However, with the advent of new technologies, long-read sequencing has made it possible to obtain complete plasmid sequences and high-resolution data on specific genomic regions. ARGs are identified using bioinformatic tools that compare genomes against constantly improving ARG databases (Djordjevic et al., 2023). Due to the rapid spread of AMR, as well as technological advances and reductions in the cost of genome sequencing, whole-genome sequencing is a powerful tool for systematic AMR surveillance.

The World Health Organization (WHO) created a Bacterial Priority Pathogens List as a tool for AMR surveillance, prevention, and control. This list includes 24 antibiotic-resistant pathogens (World Health Organization, 2024). The current study focused on Enterobacterales resistant to carbapenems and third-generation cephalosporins, vancomycin-resistant *Enterococci* (VRE), and methicillin-resistant *Staphylococcus aureus* (MRSA), as these are prevalent in livestock. Enterobacterales can carry ESBL, which confers resistance to beta-lactams, as well as genes that confer resistance to third-generation cephalosporins and carbapenems (Velasova et al., 2019; Gómez-Sanz et al., 2023). Additional resistance genes for different antibiotic classes, such as tetracycline, ampicillin, and sulfonamide, are often located on the same plasmid, leading to multidrug resistance (Lupindu et al., 2015; Shin et al., 2015; Smith et al., 2016; Velasova et al., 2019). Gram-positive *Enterococci*, such as *Enterococcus faecalis* and *E. faecium*, are members of the normal gastrointestinal flora that can cause serious infections in immunocompromised patients (Zaheer et al., 2020). Infections caused by VRE have been linked to the consumption of animal products (Arias and Murray, 2012). Vancomycin resistance is mainly mediated through acquired resistance genes (*vanA, vanB*, and *vanC)* (Wei et al., 2024). Methicillin resistance in *Staphylococcus aureus* is mediated by the mecA gene but also mecB and mecC genes, is mostly detected in pork and chicken products (González-Machado et al., 2024). MRSA also often carries plasmids with resistance genes for phenicols, lincosamides, oxazolidinones, pleuromutilins, and streptogramins (Rodrigues et al., 2020). In conclusion, surveillance of WHO priority pathogens in livestock and meat products is required to address th growing threat of antibiotic resistance. Although antibiotic use in animals in Switzerland is steadily declining, there is a need to monitor antibiotic resistance in bacterial pathogens, particularly in order to characterize ARGs using whole-genome sequencing.

Given the prevalence of antibiotic-resistant bacteria in animal food production and the risk of transmission through minimally processed RTE meat products, this study aimed to investigate the prevalence of antibiotic resistance in bacterial isolates from Swiss RTE meat products. These products were collected from artisanal and industrial butcheries in the Alpine zone and the plateau of Switzerland. Through selective plating, 156 presumptive VRE, 41 presumptive ESBL, and one MRSA isolate were obtained. The antibiotic resistance of the isolates was assessed both phenotypically and genotypically. Resistance mechanisms to clinically important antibiotics were identified, some of which were encoded on plasmids, posing a risk of rapid antibiotic resistance spread from animals to the human microbiome.

## 2 Materials and methods

### 2.1 Sample collection, sample processing, and isolation of bacteria

To gain a comprehensive understanding of antibiotic resistance in RTE meat products in Switzerland, a large-scale sampling campaign was conducted across multiple seasons to ensure that a diverse range of products was included. A total of 804 RTE meat products, including raw bacon, raw ham, Ticino dried meat, Valais dried meat, and air-dried meat produced in the canton of Grisons (Bündnerfleisch), as well as raw beef and pork sausages such as Landjäger and salami, were sampled throughout Switzerland from October 2017 to April 2019. Of these samples, 200 were purchased at the point of sale from medium-sized and large meat processing companies, while the remaining 605 were acquired directly from artisanal butchers. All samples were kept at 4 °C during transport to the laboratory.

A total of 25 g of each sample was aseptically cut and placed in a stomacher filter bag together with 225 mL of double-buffered peptone water (ISO, 2017). Each bag was then homogenized for 3 min in a stomacher (Masticator, IUL Instruments GmbH, Troisdorf, Germany) and then incubated for 24 h at 37 °C. After that, 10 μL of the enrichment suspension was spread on the following selective agar media: VRE agar, ESBL agar, Carbapenem-Resistant Enterobacterales (CRE) agar (all from Oxoid Ltd., Hampshire, UK), and MRSA agar (BioMérieux, Marcy l'Etoile, France). The *E. coli* strain ATCC 25922 was used as a negative control for ESBL and CRE plates, while the *E. faecalis* ATCC 19433 was used as the negative control for CRE agar. For positive controls, *K. pneumonia* ATCC 700603 was used for ESBL and CRE, *E. faecalis* ATCC 51299 for VRE plates, and the MRSA strain ATCC 33591 for MRSA agar plates. One typical colony per plate was transferred to Blood Agar Base plates (Oxoid) and incubated at 37 °C overnight. The isolates were then stored in 20% glycerol at −80 °C. Species identification was performed by MALDI-TOF MS using a Microflex LT/SH MALDI-TOF mass spectrometer (Bruker Daltonics, Bremen, Germany) with the MALDI Biotyper RTC Software version 3.1.

### 2.2 Antibiotic susceptibility testing

The antibiotic resistance phenotypes of the target isolates were determined based on Minimum Inhibitory Concentration (MIC) through broth dilution using the MicroScan autoSCAN-4 System (Beckman Coulter, U.S.A.). Enterobacterales isolates were tested against 31 antibiotics using the Neg MIC 44 Panel (Beckman Coulter), and *Enterococci* isolates were tested against 24 antibiotics using the Pos MIC 37 Panel (Beckman Coulter). Based on these results and the EUCAST guidelines, we selected 23 antibiotics against Enterobacterales and 10 against *Enterococci* for further analysis. The MIC values were interpreted according to the EUCAST guidelines (Version 14.0). Additionally, colistin resistance phenotypes were tested using the colistin drop test (Pasteran et al., 2020). Briefly, the colistin solution was prepared by elution of eight 10 μg colistin disks (BD, Franklin Lakes, NJ, USA) in 5 ml of cation-adjusted Mueller-Hinton broth (BD). A single 10 μL drop of the filter-sterilized 16 μg/mL colistin solution was placed on a standard agar with casein (SC) plate (BD Difco™ Plate Count Agar, Fisher Scientific) previously swabbed with an inoculum corresponding to 1.5 x 10^8^ CFU/mL of the strain. The plates were incubated for 18–20 h at 37 °C, and afterwards, the presence of inhibition zones and colonies within them were noted.

### 2.3 Short-read whole-genome sequencing

To determine the genetic mechanisms of antibiotic resistance, isolates with interesting antibiotic susceptibility profiles were subjected to whole-genome sequencing. Bacterial strains were cultured on Blood Agar Base plates (Oxoid), and genomic DNA was extracted using the GenElute kit (Sigma–Aldrich, St. Louis, U.S.A.) following the manufacturer's instructions. The concentration of DNA was determined using a Qubit^®^ 3.0 fluorometer (Life Technologies, MA, U.S.A.), and the quality was measured with a NanoDrop^®^ (Thermo Fisher Scientific, MA, U.S.A.). Standard genomic libraries were sequenced using an Illumina NovaSeq 6000 platform for paired-end reads (2 × 150 bp) by Eurofins GATC Biotech GmbH (Germany). All bioinformatics analyses were performed in Ridom SeqSphere + v10.0.0 (Jünemann et al., 2013). Accordingly, the quality of the FASTQ reads was assessed with FastQC (Andrews, 2007), and the adapters, the last 10 bp of each sequence, and sequences below a quality score of 20 were removed or trimmed with Trimmomatic (Bolger et al., 2014). For *de novo* assembly of the genomes, SKESA was used (Souvorov et al., 2018). After the quality check, we applied the Mash Identification tool to identify the species (Ondov et al., 2016) and check for contamination.

### 2.4 Long-read whole genome sequencing

For further investigation into whether the ARGs are localized on the chromosomes or plasmids, selected isolates underwent Oxford Nanopore sequencing. This technology yields long reads and makes it possible to predict the localization of the ARGs. Bacterial isolates were cultured on SC agar. Bacterial overnight cultures in the volume corresponding to 1 × 10^8^ cells in total were harvested by centrifugation (4,000 g for 2 min) and stored at −20 °C. Frozen bacterial pellets were resuspended in 400 μL TE buffer (100 mM Tris pH 8 made with TrisBase, 93362 Sigma–Aldrich, St. Louis, USA, and Tris-HCl, T3253, Sigma, 10 mM EDTA, A4892, Applichem, Darmstadt, Germany) with 15 mg/mL lysozyme (62971, Merck, Darmstadt, Germany) and incubated overnight at 37 °C. The next day, Proteinase K (final concentration 2 mg/mL, M3036, Genaxxon bioscience, Ulm, Germany), RNase A (final concentration 0.5 mg/mL, A3832, Applichem, Darmstadt, Germany), and SDS (final concentration 0.5%, 4360.1, CarlRoth, Karlsruhe, Germany) were added and incubated for 30 min at 56 °C. DNA was extracted using the Quick-DNA™ HMW MagBead Kit (D6060, ZymoResearch, Freiburg, Germany), following the manufacturer's instructions on a benchtop automated extraction instrument, the KingFisher Flex (ThermoFisher, Waltham, U.S.A.). DNA was quantified using the Pico488 dsDNA assay (Lumiprobe, Hannover, Germany). Library preparation was performed using the Rapid Barcoding Kit 96 (SQK-RBK114.96; Oxford Nanopore Technologies, Oxford, UK) according to the manufacturer's instructions. Libraries were pooled equimolarly. The final library pool was again quantified using the Pico488 dsDNA assay (Lumiprobe). Subsequently, the ONT Rapid DNA libraries were sequenced on a PromethION 2 Solo apparatus (Oxford Nanopore Technologies) using an SQK-RBK114.96 sequencing kit (Oxford Nanopore Technologies) on a FLO-PRO114M flowcell (Oxford Nanopore Technologies) running for 72 h. Raw ONT sequencing data were base-called with the super-accurate algorithm, demultiplexed, and adapter-trimmed using Dorado (version 7.3.11; Seymour, 2024). Long reads in FASTQ format were quality-checked using NanoPlot (version 1.42.0, De Coster and Rademakers, 2023) and filtered using Filtlong (version 0.2.1, Wick, 2021), discarding reads with an average quality below Q10 or a length below 1000 bp. The surviving long reads were assembled into contigs using Flye (version 2.9.3; Kolmogorov et al., 2019). Contigs were polished with the same long reads using Racon (version 1.5.0; Vaser et al., 2017) and Medaka (version 1.12.1; Wright, 2024). Further polishing was performed with Illumina short reads using Pilon (version 1.24; Walker et al., 2014) and Polypolish (version 0.6.0; Wick and Holt, 2022). Taxonomic identity and annotation of the polished contigs were determined with PGAP (version 2024-07-18.build7555; Tatusova et al., 2016).

### 2.5 Detection of resistance genes, mobile genetic elements, and sequence typing from whole-genome sequencing data

All the bioinformatic analyses to identify resistance genes and mobile genetic elements (MGEs) were performed in the SeqSphere software (Jünemann et al., 2013). The tool AMRFinder Plus was used to find ARGs or genes related to biocide and metal resistance (BMRGs) from the NCBI Bacterial Antimicrobial Resistance Reference Gene Database (Feldgarden et al., 2019). AMRFinder Plus classifies the beta-lactam resistance genes in five different subclasses: beta-lactam, carbapenem, cephalosporin, cephalothin, and methicillin. We would like to highlight that, although beta-lactam is not a subclass itself, we kept this nomenclature to group the genes that did not belong to any other subclass; thus, we classified them here as penicillin resistance genes. To identify MGEs in WGS sequence data, the Mobile Element Finder was also applied (Johansson et al., 2021). For reconstruction and typing of the plasmids, the MOB-suite was used (Robertson and Nash, 2018).

### 2.6 Molecular typing of selected bacterial isolates

The SeqSphere software was also used to type selected bacterial isolates for which the multi-locus sequence typing (MLST) scheme was available, such as *E. faecium* (Homan et al., 2002), *E. faecalis*, and *S. aureus* (Enright et al., 2000). For *S. aureus*, spa typing was performed additionally (Harmsen et al., 2003; Mellmann et al., 2007).

### 2.7 Localization of resistance genes

To determine the location of ARGs, we used SourceFinder to predict whether the sequences originated from chromosomes, plasmids, or bacteriophages (Derya et al., 2022). The SourceFinder tool classifies these elements based on k-mer distributions of complete and partial genome sequences and is available as an online web service.

### 2.8 Data management and visualization

Multi-drug resistance (MDR) was defined as resistance to at least one antibiotic from three or more classes (Magiorakos et al., 2012). The resistance rate was calculated as n(strain.res)/n(strain.tot), where n(strain.res) denotes the number of strains resistant to a given antibiotic tested and n(strain.tot) the total number of strains tested. The Multiple Antibiotic Resistance (MAR) index describes how many of the tested antibiotics to which a strain is resistant. It was calculated for each strain across all tested antibiotics using the formula *n*(antibiotic-resistant isolates)/*n*(total antibiotics) (Krumperman, 1983). The correlation between the presence of ARGs and MRGs was assessed using Pearson's correlation coefficient. We used *R* version 4.4.1 (R Core Team, 2024) for statistical analysis and visualization of the data. We used the following packages for data analysis and visualizations: tidyverse (Wickham et al., 2019), readxl (Wickham and Bryan, 2023), knitr (Xie, 2024), BiocManager (Morgan and Ramos, 2024), devtools (Wickham et al., 2022), remotes (Csárdi et al., 2024), htmlwidgets (Vaidyanathan et al., 2023), and ggbeeswarm (Clarke et al., 2023).

## 3 Results

### 3.1 Different potentially pathogenic bacteria isolated from ready-to-eat meat products

To investigate the prevalence of ARB in RTE meat products, we isolated bacteria from 804 different products using different selective media targeting VRE and ESBL-producing Enterobacterales. Overall, we recovered 198 isolates (Supplementary Figure 1) that were identified using MALDI-TOF as presumptive VRE [*E. faecalis* (*n* = 146), *E. faecium* (*n* = 10)] and 41 as presumptive ESBL-producers or carbapenem-resistant Enterobacterales, namely *Citrobacter* (*C. freundii* and *C. braakii*), Enterobacter (*E. cloacae, E. hormaechei*, and *E. kobei*), *Proteus* (*P. hauseri* and *P. vulgaris*), and *Escherichia coli*, and one MRSA isolate.

### 3.2 Several bacterial isolates exhibit resistance to critical antibiotics

**Figure 1:**
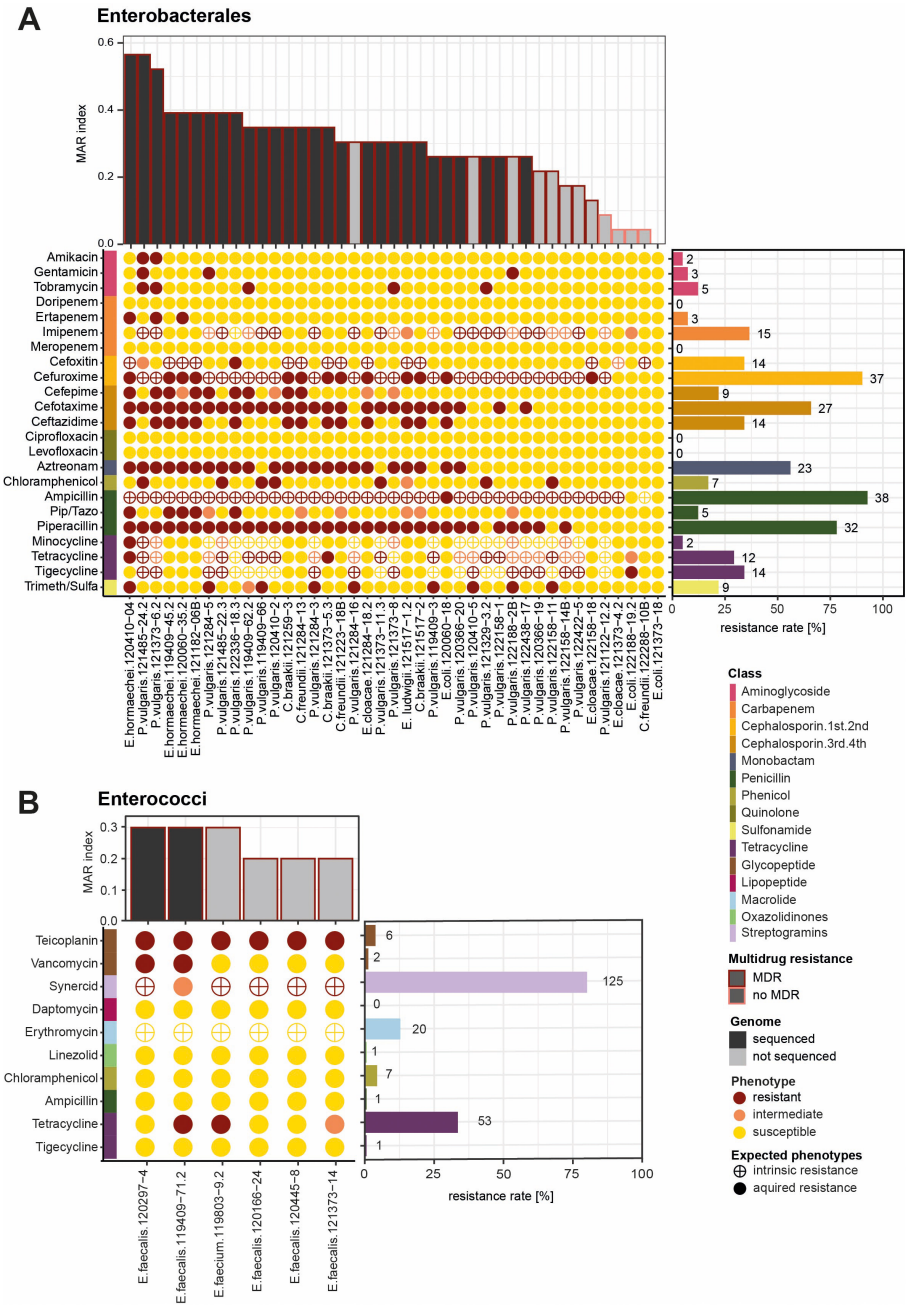
Antibiotic resistance phenotype of Enterobacterales and *Enterococci*: **(A)** The bar graph at, the top shows the MAR index of the bacterial isolates belonging to Enterobacterales (*n* = 41). Bars colored in black mark the isolates that were whole-genome sequenced, while the colored borders indicate if they are MDR. The color of the dots represents the phenotype of each bacterial isolate in response to the tested antibiotics, classified as resistant, intermediate, or susceptible. In the colored bar on the left side, antibiotics are annotated according to their class. The bar graph on the left shows the resistance rate calculated by the proportion of all isolates being resistant to a given antibiotic. **(B)** The same plots for a selection of six *Enterococci* isolates. The resistance rates were calculated from the total number of *Enterococci* isolates tested in this study (*n* = 156).

**Figure 2:**
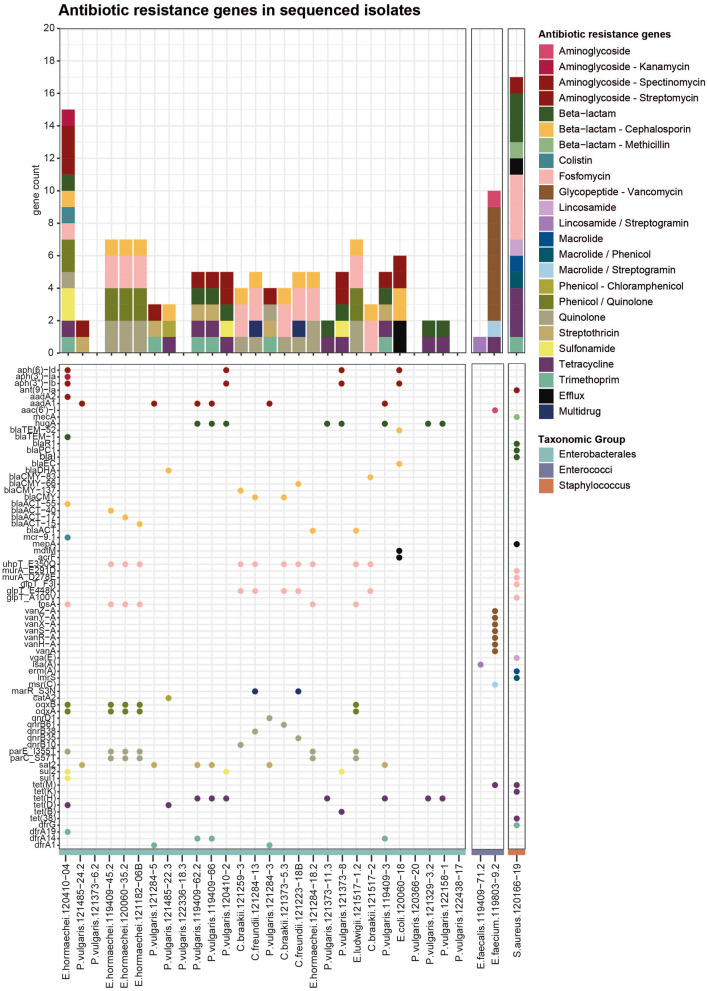
Antibiotic resistance genes in bacterial isolates from meat: Identified using AMRFinder Plus on short-read sequencing data. The upper bar graph shows all ARGs per strain, colored by antibiotic class. The lower dot plot represents all single genes (listed on the y-axis), and dots are colored according to the antibiotic class.

Antibiotic resistance profiles of the purified bacterial isolates from meat were assessed using the semi-automated system MicroScan. For the gram-negative Enterobacterales, we tested 23 antibiotics belonging to 12 different classes. Among the 41 tested Enterobacterales (*Proteus, Enterobacter, Citrobacter* and *Escherichia*), 40 strains were resistant to at least one antibiotic and 36 were resistant to one antibiotic from 3 or more antibiotic classes and thus considered as multidrug resistant (MDR) (Figure 1). The MAR index was calculated for each strain among all tested antibiotics and describes how many of the tested isolates a strain is resistant to Krumperman (1983). In total, 23 isolates had a MAR index greater than 0.3, indicating that they were resistant to 30% of the tested antibiotics. Three Enterobacterales (one *E. cloacae* and two *P. vulgaris*) had a MAR index higher than 0.5. Enterobacterales belonging to the genera *Proteus, Citrobacter, and Enterobacter*, and the species *E. coli* resistant to third- and fourth-generation cephalosporins, and *Enterobacter* and *E. coli* resistant to carbapenems are considered priority pathogens by WHO. We identified many strains resistant to cephalosporins from the third generation (27 cefotaxime and 14 ceftazidime resistant) and the fourth generation (9 cefepime) (Figure 1). Namely, we detected 5 *Citrobacter* strains, 6 *Enterobacter* and 1 *Escherichia* resistant to the third-generation cephalosporins tested (cefotaxime and ceftazidime) and 14 *Proteus* isolates resistant to cefotaxime but susceptible to ceftazidime. Resistance to the second-generation cephalosporin cefuroxime was observed in 14 strains, while *Proteus* isolates are intrinsically resistant to cefuroxime and cefoxitin. Carbapenem resistance was rare; only three strains were resistant to ertapenem, namely 2 *Enterobacter* isolates and one *Proteus*. For the penicillins, 32 strains were resistant to piperacillin, but only five strains (all *E. cloacae*) were resistant to the combination of piperacillin with tazobactam. One *E. cloacae* and one *C. braakii* isolate showed resistance to tetracycline, while *Proteus* are intrinsically resistant to tetracyclines. One *Enterobacter* and 8 *Proteus* isolates were resistant to the combination of trimethoprim and sulfamethoxazole. Resistance to the monobactam aztreonam was detected for 23 isolates. Resistance to aminoglycosides was rare (2 resistant to amikacin, 3 to gentamicin, and 5 to tobramycin). Five *Proteus* isolates were resistant to chloramphenicol. All tested isolates showed resistance to fosfomycin (Supplementary Figure 2), though EUCAST discourages the susceptibility testing for this antibiotic due to its tendency to degrade in certain media or conditions. Accordingly, fosfomycin resistance is not discussed further here. No resistance against quinolones was detected. The positive results for last-resort antibiotic colistin were confirmed with a colistin drop test for all the taxa not intrinsically resistant. In total, two isolates, one *E. cloacae* and one *E. coli* isolate, were colistin resistant (Supplementary Figure 3). For the gram-positive *Enterococci* (*E. faecalis* and *E. faecium*), resistance against 10 antibiotics belonging to 8 antibiotic classes was assessed (Figure 1B, Supplementary Figure 4). Among the 156 tested *Enterococci*, 137 strains were resistant to at least one antibiotic, and 112 were resistant to one antibiotic from 3 or more antibiotic classes and thus considered MDR. Over 25% of the isolates tested were resistant to tetracycline. Both *E. faecium* and *E. faecalis* are intrinsically resistant to the streptogramin antibiotic synercid and the macrolide erythromycin. Among all isolates tested, 112 were multidrug resistant (Supplementary Figure 4). For five isolates, the MAR index was greater than 0.3, whereas 17 isolates showed a MAR index higher than 0.2. Still, 18 isolates were susceptible to all antibiotics tested. We identified two *Enterococci* isolates resistant to the glycopeptide antibiotic vancomycin, one *E. faecium* and one *E. faecalis* (Figure 2). The vancomycin-resistant *Enterococcus faecium* isolate 119803-9.2 was identified as Sequence Type (ST) 133, part of clonal complex 17 (CC17), and classified within clade A. *E. faecium* clade A includes clinical isolates (A1) and animal-associated isolates (A2), while clade B comprises fecal isolates from healthy humans (Wei et al., 2024). As ST133 is associated with clade A2, primarily linked to animal sources (Biggel et al., 2021), isolate 119803-9.2 likely originated from an animal reservoir. Seven isolates were resistant to the other tested glycopeptide antibiotic, teicoplanin. A few strains show resistance against chloramphenicol (*n* = 7), the oxazolidinone linezolid *(n* = 1), and ampicillin (*n* = 1). Another WHO priority pathogen detected in our study was MRSA. Apart from resistance to methicillin (oxacillin), the *S. aureus* isolate also showed phenotypic resistance to penicillin and ampicillin, the macrolides azithromycin and erythromycin, tetracycline, the lincosamide clindamycin, gentamicin, and fusidic acid (Supplementary Figure 5). MLST typing classified this *S. aureus* isolate to belong to the clonal complex 398, which is the typical livestock-associated MRSA (Price et al., 2012). Further spa typing identified the strain as t034, which is associated closely with porcine strains usually harboring more resistance genes and acquired immune evasion gene clusters (Kittl et al., 2020). In our study, we identified 53 distinct bacteria-antibiotic resistance combinations, where different pathogens exhibited resistance to specific antibiotics, highlighting diverse pairings of pathogens and their resistance mechanisms present in RTE meat.

### 3.3 The presence of antibiotic resistance genes partially explains resistant phenotypes

To identify the genetic determinants underlying the observed antibiotic resistance phenotypes, 31 selected isolates were subjected to whole-genome sequencing using Illumina short-read technology. Isolates were chosen based on their phenotypic resistance profiles. Among the Enterobacterales, 28 strains were selected, most of which had a MAR index greater than 0.25, of which three strains displayed primarily intrinsic resistance to some of the tested antibiotics (Figure 1A). Additionally, two *Enterococcus* strains displaying phenotypic resistance to vancomycin and the MRSA strain were included. Screening the resulting genome assemblies with AMRFinderPlus identified 164 distinct ARGs across 25 classes (Figure 2). The most prevalent resistance gene classes were those conferring resistance to fosfomycin, aminoglycosides, quinolones, tetracyclines, and cephalosporins. ARGs were detected in 24 out of 28 Enterobacterales isolates. Notably, genes associated with cephalosporin resistance were identified in 13 strains (Figure 2), predominantly encoding AmpC beta-lactamases, such as *blaDHA* in *Proteus*, various variants of *blaCMY* in *Citrobacter*, multiple variants of *blaACT* in *Enterobacter*, and *blaEC* in *E. coli*. Only one ESBL was identified in the *E. coli* isolate, namely *blaTEM-52*, but it was not confirmed by long-read Nanopore sequencing later on, either because the plasmid was lost between the sequencing events or due to issues in the assembly. Additionally, two beta-lactamases not categorized as AmpC or ESBL were identified, namely the broad-spectrum class A beta-lactamase *hugA* in several *Proteus* isolates and the *blaTEM-1* in one *Enterobacter*. No specific carbapenem resistance genes were detected, including in the three strains exhibiting ertapenem resistance. Five strains carried multiple resistance genes, including the *blaACT* beta-lactamase conferring cephalosporin resistance, the fosfomycin resistance gene *fosA* encoding a glutathione transferase, multidrug efflux RND transporters *oqxA*/*oqxB* associated with phenicol and quinolone resistance, and quinolone resistance proteins with mutations (*parE_I355T, parC_S57T*). Another resistance pattern observed in four strains involved aminoglycoside resistance genes (*aadA1*/*aadA2*), the penicillin resistance gene *hugA*, the streptothricin resistance gene *sat2*, tetracycline efflux MFS transporters (*tet(H)*/*tet(D)*), and trimethoprim resistance genes (*dfrA1, dfrA14, dfrA19*) (Figure 2). Sulfonamide resistance genes (*sul1*/*sul2*) were identified in *Proteus vulgaris* and *Enterobacter cloacae*. In *P. vulgaris*, the chloramphenicol resistance gene *catA2* (encoding an O-acetyltransferase) was detected in one isolate, while the streptothricin resistance gene *sat2* (an N-acetyltransferase) was present in five isolates. Only the *E. coli* strain harbored two multidrug efflux transporters, *acrF* and *mdtM*. In the multidrug-resistant *E. cloacae* isolate 120410-04, a resistance gene was identified for each observed antibiotic resistance phenotype, including beta-lactam (*blaTEM-1*), cephalosporin (*blaACT-55*), fosfomycin (*fosA*), colistin (*mcr* 9.1), tetracycline (*tetD*), trimethoprim (*dfrA19*), and sulfonamide (*sul1* and *sul2*). Interestingly, three *E. hormaechei* strains, which were isolated from different products and distinct sampling points, carried the same set of chromosomally encoded resistance determinants, including quinolone resistance mutations in *parE* and *parC*, phenicol/quinolone resistance genes *oqxA* and *oqxB*, fosfomycin-associated mutations in *uhpT*, the fosfomycin resistance gene *fosA*, and the beta-lactamase gene *blaACT*. This pattern suggests that certain resistant strains may be present across multiple products and producers over time, indicating possible recurrence within the wider production or distribution network.

For the *Enterococci*, the typical vancomycin-resistance gene cassette, including *vanA* linked to other *van* genes, was only detected in the *E. faecium* isolate 119803-9.2 (Figure 2), located in the Tn*1546* transposon. Furthermore, the tetracycline resistance gene *tet(M)*, the aminoglycoside acetyltransferase *aac6*′*)-I*, and the macrolide/streptogramin resistance gene *msr(C)*. *E. faecalis* isolates harbored only a resistance gene for lincosamides and streptogramin, namely the gene *lsa(A)* encoding the ribosomal protection protein lincosamide/streptogramin. In the MRSA isolate, the characteristic *mecA* gene was detected. Further resistance genes for penicillin (*blaI, blaPC1* and *blaR1*), tetracycline *tet(38), tet(K)* and *tet(M)*, trimethoprims (*dfrG*), macrolides (*erm(A)*), lincosamides (*vga(E)*) and aminoglycosides (*ant(9)-la*) were identified. Additionally, the strain had one multidrug efflux pump (*mepA*) and was found to carry point mutations in four different genes, all conferring resistance to fosfomycin. These results underscore a broad spectrum of resistance mechanisms encoded in the genomes of bacterial isolates from Swiss meat products.

To test how genetic factors contribute to observed antibiotic resistance, the correlation between resistance phenotypes and the presence of ARGs was analyzed (Supplementary Figure 6). When the resistance gene was present for beta-lactams, cephalosporins, tetracyclines, sulfonamides, and trimethoprim, the isolate was phenotypically resistant in most cases. A strong correlation was also observed for fosfomycin; however, as previously mentioned, the topic of fosfomycin resistance is not discussed further here due to technical limitations. Fewer matches were detected for aminoglycosides and phenicols. This indicates that the presence of known resistance genes explains much of the observed antibiotic resistance. However, some resistance phenotypes may involve other mechanisms not captured by the current genetic analysis.

### 3.4 Biocide and metal resistance genes co-occur with antibiotic resistance genes

**Figure 3:**
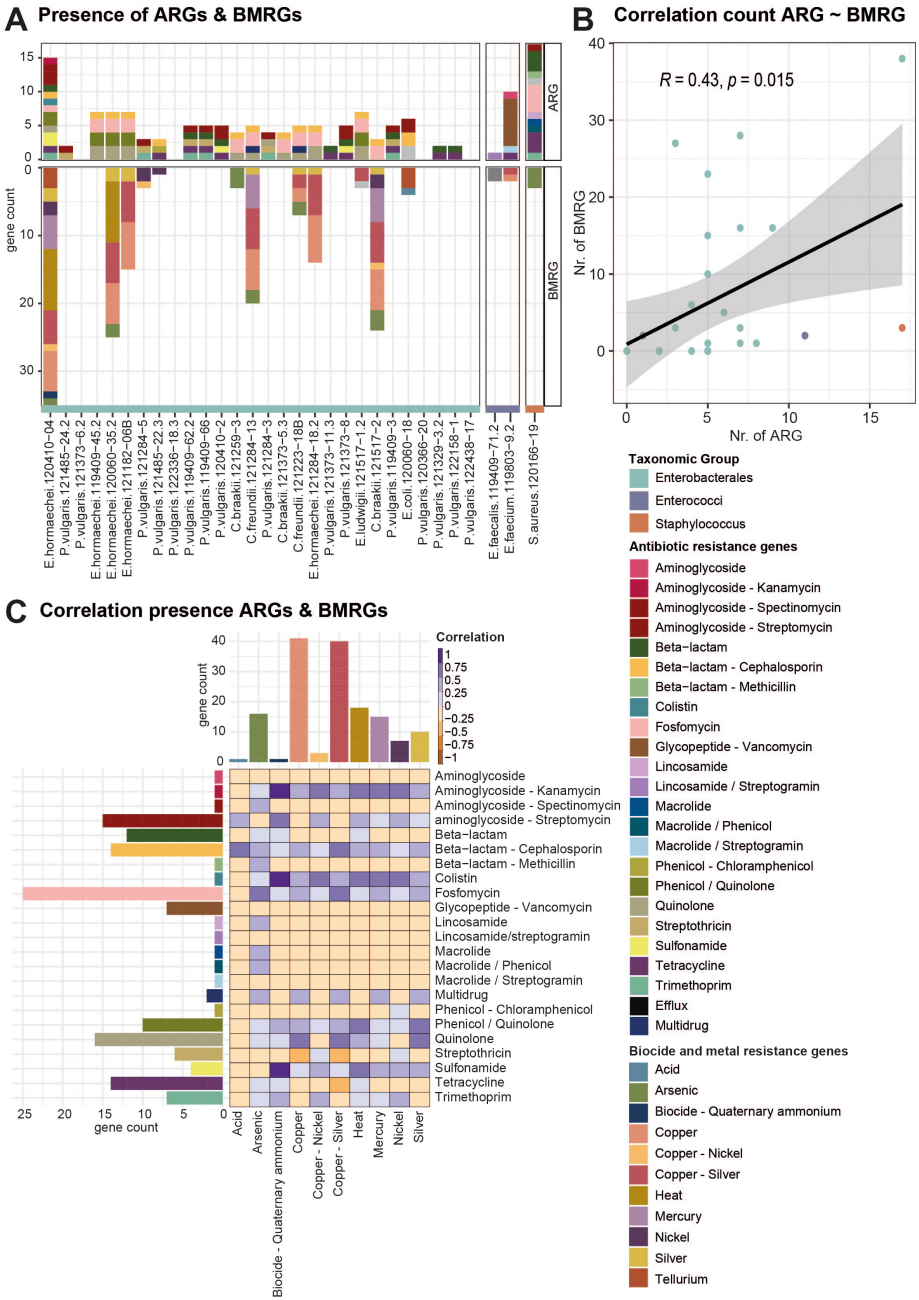
Correlation of the presence of ARGs with BMRGs. **(A)** Bargraph displays all ARGs and BMRGs detected in all isolates by short-read sequencing. Bars are colored according to the antibiotic or biocide/metal resistance class. **(B)** Scatter plot showing the correlation of the count of ARGs with BMRGs per strain. Dots are colored according to the taxonomic groups of the strains. **(C)** Correlation of the presence and absence of different classes of ARGs and BMRGs. High correlation is indicated with a dark purple color, while negative correlation indices are colored in orange. Bar graphs on the side indicate the count of ARGs and BMRGs per class.

Non-antibiotic agents such as biocides or heavy metals in the environment can co-select for ARGs (Murray et al., 2024). Thus, we also profiled the presence of BMRGs in all strains (Figure 3A, Supplementary Figure 7). Genes annotated as BMRGs, including resistance to heat, acid, biocides, and heavy metals, were found in half of the sequenced strains (15/31). In six of the strains, genes conferring resistance to copper and silver were present; in five of them, arsenic resistance genes were present. Further resistance genes for mercury (3 strains), nickel (4 strains), copper and nickel (3 strains), and heat (3 strains) were detected. Genes for resistance against quaternary ammonium compounds, acid, bacitracin, and efflux pumps were detected only in one strain each. In the Enterobacterales strains, the number of BMRGs correlated with the number of ARGs (Pearson product-moment correlation, *p*-value = 0.015, coefficient = 0.43, Figure 3B). BMRGs were only identified in strains that also carried ARGs and not in strains that did not carry ARGs. Several positive correlations exist between certain AMR classes and stress, but the data must be interpreted with caution as it is a small dataset and the number of genes varies considerably, affecting the relevance of the correlations (Figure 3C, Supplementary Table 5). Significant positive correlations exist between quinolone resistance genes and copper (15 genes) and between quinolone and copper/silver resistance genes (40 genes). The co-occurrence of ARGs and BMRGs points to the potential co-selection of ARGs through stressors such as copper or silver.

### 3.5 Prediction of localization and mobilization of ARGs

**Figure 4:**
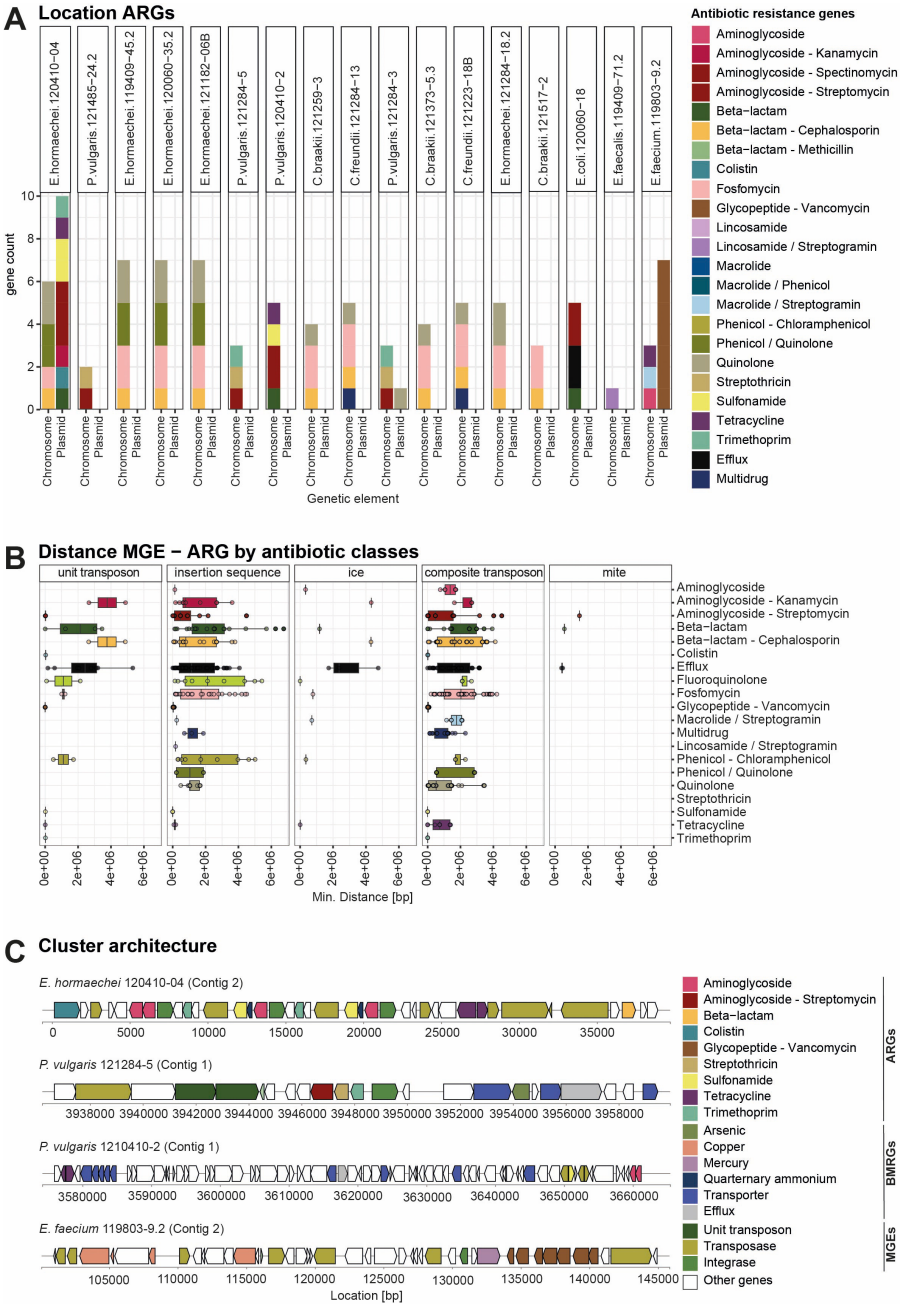
Localization of ARGs on the chromosome and MGEs based on long-read sequencing data. **(A)** A bar graph showing the count of ARGs per strain and their location on the chromosome or plasmid. The bar is colored according to the class of ARGs. **(B)** Boxplot visualizing the minimal distance between each ARG, grouped by class, and the next MGE in the chromosome. Ice, integrative and conjugative elements; mite, miniature inverted-repeat transposable elements. MGEs were identified using MobileElementFinder. **(C)** Cluster architecture of selected strains based on the annotation from hybrid genome assembly from short- and long-read sequences. Cluster plots show the proximity of different ARGs to MRGs and MGEs.

An important aspect for the dissemination of ARGs in the environment is the mobility of the genes. ARGs are often transferred by MGEs such as plasmids or transposons located in the chromosomes from one bacterial genome to another (Partridge et al., 2018). To investigate whether ARGs are located in the chromosome or on a plasmid, we applied long-read nanopore sequencing. Using the SourceFinder prediction tool (Derya et al., 2022), we found that most ARGs were localized on chromosomes and not on plasmids (Figure 4A). Only vancomycin resistance genes in *E. faecium* 119803-9.2 were clearly localized on a plasmid, likely a conjugative plasmid as predicted by Mobile Element Finder (Figures 4A, C, Supplementary Figure 8). To investigate the potential mobility of the genes located on the chromosome, we calculated the minimal distance of each antibiotic resistance gene to the closest mobile genetic element (Figure 4B). Genes conferring resistance to colistin, sulfonamide, trimethoprim, tetracycline, streptomycin, streptothricin, and vancomycin showed a minimal distance of 0 bp, indicating their location on MGEs such as unit and composite transposons or insertion sequences. The close location of the different resistance genes and MGEs in the genome is visualized in the cluster plots in Figure 4C. *E. hormaechei* 120410-04 harbors a conjugative plasmid and was classified to the incompatibility group IncHI2A. This plasmid carries the *mcr9.1* gene, which confers colistin resistance, and is closely located to an IS26 family transposase. Shortly downstream there is a region containing aminoglycoside resistance genes (*aph(6)-Id, aph(3*^′′^*)-Ib*), followed by a region with a duplicated sequence of genes containing trimethoprim (*dfrA19*) sulfonamide resistance genes (*sul1*) and a quaternary ammonium efflux transporter (*qacE*) gene flanked by a class 1 integrons (*intI1*)and an IS26 family transposase. Further downstream, there are two tetracycline resistance genes (*tetD, tetR*) next to an IS26 family transposase. Further downstream, a beta-lactamase (*blaTEM-1*) is located close to a Tn*3* family transposase. In *P. vulgaris* 121284-5, the resistance genes for streptomycin, streptothricin, and trimethoprim are located next to each other, flanked by an integrase and closely located next to a unit transposon. On the conjugative plasmid from *E. faecium* 119803-9.2, the vancomycin resistance gene cassette is located next to a mercury resistance gene and flanked by an integrase and a transposase. Close to this region, several copper resistance genes are present. We further predicted the genomic localization of BMRGs, revealing that a greater proportion of BMRGs were likely plasmid-borne compared to ARGs (Supplementary Figure 9). Even though the majority of the ARGs are located on chromosomes rather than plasmid-borne, they have potential mobility because they are closely located to MGEs on the chromosome.

## 4 Discussion

This study investigated the presence of ARB and ARGs in Swiss RTE meat products. Switzerland, like other European countries, has introduced measures that lead to a reduction in antibiotic use in livestock production (Bundesamt für Lebensmittelsicherheit und Veterinärwesen BLV, 2024). However, various practices throughout the food chain contribute to the cross-contamination of meat products with ARB products (Cobo-Díaz et al., 2021; Barcenilla et al., 2024). Additionally, non-antibiotic compounds, including heavy metals and biocides, can co-select ARB along the food chain (Murray et al., 2024). As our study is, to the best of our knowledge, the first to report ARB in RTE meat products in Switzerland, and we did not evaluate the prevalence of antibiotic resistance systematically, direct comparisons with other studies are challenging. Over the food chain, particularly in uncooked foods such as RTE meat products, resistant pathogens and resistance genes can be transmitted to humans and thus present a public health issue (Jans et al., 2018). Below, we discuss the prevalent types, the selection, and the implications for public health of antibiotic resistance in RTE meat products.

### 4.1 Antibiotic resistance in pathogens from ready-to-eat meat products

Even though the use of antibiotics in Switzerland is declining gradually, 79% of all antibiotics used are still administered to animals. The most commonly used antibiotics in Swiss livestock farming are penicillins, tetracyclines, sulfonamides, and aminoglycosides (Bundesamt für Lebensmittelsicherheit und Veterinärwesen BLV, 2024; Federal Office of Public Health and Federal Food Safety and Veterinary Office, 2024). A recent meta-analysis conducted in Switzerland identified raw meats, milk, seafood, and certain fermented dairy products as the highest-risk sources of ARB, with medium to high exposure levels (Jans et al., 2018). Compared to raw plant-based or raw milk products, gram-negative and gram-positive foodborne pathogens and indicator bacteria - such as Enterobacterales, *E. coli, Enterococcus*, and *Staphylococcus*—were predominantly found in raw meat products, with much lower levels detected in raw plant products (Jans et al., 2018). In our study, we detected mainly multidrug-resistant Enterobacterales, two vancomycin-resistant *Enterococci*, and one MRSA. We identified several ARB, including multidrug-resistant strains and pathogens from the WHO priority list. Overall, we detected multidrug-resistant bacteria in 18% of the samples. In our study, most of the cephalosporin-resistant Enterobacterales expressed the common phenotype associated with the harboring of an AmpC beta-lactamase, being resistant to penicillins, cephalosporins, and cefoxitin. Only one ESBL was detected in one *E. coli* strain, although it was simultaneously present with one AmpC beta-lactamase. These results are consistent with previous studies that demonstrated how meat products can be a reservoir of different ARB. In a study done on Swiss chicken meat, the prevalence of ESBL was found to be 25.9% (Gómez-Sanz et al., 2023). Enterobacterales resistant to third-generation cephalosporins and carbapenems were also found in the meat production chain (Garofalo et al., 2024). Although antibiotic-resistant Enterobacterales have been found in raw meat, there is limited data on RTE meat products (Zarzecka et al., 2022). Regarding the *Enterococci*, it is noteworthy that out of 156 isolates recovered from VRE agar, only two were confirmed to be phenotypically resistant to vancomycin, and only one was a carrier of vancomycin resistance genes. This suggests that many vancomycin-susceptible isolates grew on the VRE agar, indicating its weak specificity in selecting VRE. The low percentage of *Enterococci* that were resistant to vancomycin, in contrast to the high percentage of them being resistant to tetracycline, was already reported in food samples (Pesavento et al., 2014). Similarly, multidrug-resistant *Enterococci* isolates were found in 74.1% of the tested RTE meat products, but none of the isolates were resistant to vancomycin (Chajecka-Wierzchowska et al., 2016). Conversely, percentages of VRE are usually higher in raw meat products, such as a previous study that showed how 23.8% of the tested meat products from Spain carried VRE (Guerrero-Ramos et al., 2016). The single MRSA isolate we found agrees with the low detection of MRSA in other studies, such as the 2% prevalence in RTE meat products from pork in the Czech Republic (Gelbíčová et al., 2022). In contrast, in another study performed in Egypt, MRSA was identified in 23.4% of the RTE meat products (Saber et al., 2022). Our present study, where 24% of the samples were positive for ARB, addressed the potential knowledge gap on the presence of ARB in fermented and cured meat products as highlighted by Jans and co-authors (Jans et al., 2018). The presence of various multidrug-resistant and well-known human pathogens in RTE meat products highlights their potential as a reservoir for spreading pathogenic and antibiotic-resistant bacteria from animals to humans.

### 4.2 Origin of antibiotic resistance

Contamination of RTE meat products with ARB can occur through multiple pathways. Raw meat's slightly acidic pH and high water activity create an ideal environment for bacterial growth (Nychas et al., 2008). Meat contamination often occurs during slaughter from the hide, gastrointestinal tract, and skin (Weinroth et al., 2022), but it can also be transmitted from the environment during food processing (Cobo-Díaz et al., 2021; Barcenilla et al., 2024). The focus of our study was to assess the occurrence and diversity of ARB in RTE meat products from various Swiss butcheries, rather than to identify contamination sources. Sequence typing of the VRE and MRSA isolates indicates an animal origin. The VRE isolate belongs to clade A2, which is associated with animal sources (Wei et al., 2024) and may act as a reservoir for vancomycin resistance despite limited immediate risk to human health. This is supported by previous findings of ST133 isolates in Swiss aquatic environments, suggesting environmental transmission (Biggel et al., 2021). The MRSA strain belongs to clonal complex CC398, typical of livestock-associated MRSA (LA-MRSA), specifically spa t034, known for multiple resistance genes and immune evasion clusters (Price et al., 2012; Kittl et al., 2020). LA-MRSA causes infections in animals and humans, with pigs as a primary reservoir where MRSA prevalence is rising in Switzerland (van Loo et al., 2007; Kittl et al., 2020). Since no MLST typing scheme is available for the Enterobacterales isolated here, it is not possible to determine whether contamination originated from animals or the food processing environment. However, several studies have shown that Enterobacterales carrying various ARGs, as well as VRE, commonly contaminate dry-aged, cured, fermented, and cut meat products during processing (Cobo-Díaz et al., 2021; Barcenilla et al., 2024). Interestingly, we identified three *E. hormaechei* strains in our study that carried an identical set of chromosomally encoded resistance determinants (quinolone, phenicol/quinolone, fosfomycin, and beta-lactamase), suggesting that certain resistant strains may persist across multiple products and producers over time, indicating potential re-occurrence within the broader production or distribution network. Future work is needed to identify potential sources of ARB, analyze transmission routes, and identify factors increasing persistence in food-producing environments.

### 4.3 Correlation of the AMR phenotype with AMR genotype

The antibiotic resistance phenotypes of the isolates only correlate weakly with the presence of ARGs (Supplementary Figure 6). The most consistent resistance patterns were observed for beta-lactams and tetracyclines. The lack of correlation between the antibiotic resistance phenotypes and genotypes is very well-known in microbiology and can be attributed to several factors. One explanation is that some ARGs are not expressed, leading to a phenotypic susceptibility despite the presence of the resistant genotype (Kime et al., 2019). For example, several *P. vulgaris* strains have been found to have the aminoglycoside resistance genotype but do not display phenotypic resistance, which could be due to mutations in promoter regions affecting the phenotype (Davis et al., 2011). Another reason is the presence of nonspecific resistance mechanisms such as efflux pumps, which could confer resistance to several different antibiotics but are not classified as such (Nishino et al., 2021). In our study, we identified the *acrF* gene in *E. coli*. The efflux pump acrEF was reported to lead to increased resistance to fluoroquinolones and other drugs in *E. coli* (Zhang et al., 2018). The *oqxAB* genes, which were detected here in several MDR isolates, encode for Resistance-Nodulation-Cell-Division (RND) family efflux pumps and mainly confer resistance to several broad-spectrum antibiotics. The overexpression of efflux pumps like oqxAB can also lead to decreased susceptibility to structurally unrelated antibiotics such as aztreonam due to extrusion (Bialek-Davenet et al., 2015). Also, mutations in regulatory regions can lead to overexpression of *oqxAB*, increasing antibiotic resistance to multiple antibiotics (Dawson et al., 2024). Apart from mechanisms such as gene regulation and nonspecific efflux that affect the phenotype, methodological issues in genome sequencing for the detection of resistance genes contribute to discrepancies between phenotype and genotype. One reason is that the identification of resistance genes by WGS relies on databases, and when they are incomplete or outdated, they can lead to missed or incorrect annotations (Feldgarden et al., 2021). Other reasons are that WGS may not detect resistance conferred by regulatory mutations or alterations in gene expression (Sun et al., 2019). Furthermore, novel or rare mutations may not be well annotated for their role in resistance. Moreover, the genome assembly could be interrupted and lead to the missing identification of resistance genes, as recently shown for metagenomic assemblies (Abramova et al., 2024).

Furthermore, the choice of guidelines used to classify antibiotic resistance may also influence the results. In this study, we followed the EUCAST guidelines, which primarily focus on clinical isolates. Other guidelines, such as those from the Clinical and Laboratory Standards Institute (CLSI, https://clsi.org/), also include thresholds for environmental species. Incorporating these additional criteria could help provide a more comprehensive correlation between phenotypic and genotypic resistance. Together, this shows that gene regulation and nonspecific efflux mechanisms can affect phenotypic resistance of bacteria to different antibiotic classes, but also that methodological issues are leading to inaccurate identification of resistance genes. These findings highlight that phenotypic and genotypic methods need to be combined for the monitoring of antibiotic resistance.

### 4.4 Co-selection through different stressors

In addition to antibiotics, non-antibiotic agents such as metals, biocides, acids, or heat stress can also co-select for antibiotic resistance in the food chain (Murray et al., 2024). Heavy metals such as copper, zinc, or cadmium, commonly used in agriculture and animal livestock, can select for bacteria that carry BMRGs and ARGs (Rensing et al., 2018; Gillieatt and Coleman, 2024). Zinc and copper are commonly used as livestock feed supplements for infection control and growth promotion (Yue et al., 2021). Even though the use of heavy metals in Switzerland is nowadays regulated, recent data indicate that high amounts of copper and zinc are present in Swiss soils (Gross et al., 2021), which are linked to the fertilization using farmyard manure originating from livestock farming. Also, arsenic, mercury, and silver have been used as antimicrobials in veterinary medicine for decades (Argudín et al., 2019). Studies have shown that heavy metals such as copper, zinc, and arsenic are found in high concentrations in soils, particularly in soils fertilized with manure, where they persist and accumulate over time (Ji et al., 2012). The bacterial isolates from this study showed a positive correlation between the number of ARGs and BMRGs, indicating the potential for metal co-selection (Figure 3B). This aligns with previous research demonstrating that bacteria with BMRGs are more likely to also carry ARGs compared to those without BMRGs. Clinically important genera from humans and animals, such as *Staphylococcus*, were found to harbor the highest numbers of both ARGs and BMRGs (Pal et al., 2015). ARGs and BMRGs are often found in close proximity in bacterial genomes (Pal et al., 2015; Li et al., 2017). In our study, which focused on the prevalence of ARG in RTE meat products, we did find similar patterns including the co-occurrence of the BMRGs for arsenic, copper, mercury and silver with the ARGs for beta-lactams/cephalosporins, quinolones, sulfonamides and aminoglycosides (Figure 4A). Even though the frequent co-occurrence of ARGs and BMRGs observed in genomic studies indicates the potential for indirect selection of ARG by metals leading to co-resistance, there is limited knowledge on the mechanisms acting in the environment and also how the phenotypic resistance will be expressed (Gillieatt and Coleman, 2024). Several studies have confirmed experimentally that metals and biocides can select for ARGs through cross-resistance (Gillieatt and Coleman, 2024; Murray et al., 2024). For example, the AcrAB-TolC efflux pump can pump out of the cell multiple antibiotics and biocides and non-antibiotic drugs (Murray et al., 2024).

Furthermore, efflux pumps may exhibit broader specificity than their annotations suggest (Gillieatt and Coleman, 2024). The implications of co-selection by metals and biocides for antibiotic resistance in the environment, the concentrations of the relevant compounds that are selective (including sub-MIC and residuals), and the likely mechanisms still need to be investigated. Furthermore, ARG databases mostly contain genes identified in clinical isolates after exposure to antibiotics. Non-antibiotic agents could select for new resistance genes that have not been annotated and that arise from the environment, posing an additional risk of transfer to human, animal, and plant pathogens. Given the use of metals in livestock and their potential for co-selection, monitoring metal use and resistance in agriculture could help reduce the spread of AMR from animals to humans.

### 4.5 Potential of mobility of ARGs

ARGs can be located on the bacterial chromosomes or plasmids. Their location depends on evolutionary dynamics and selective pressures (Harrison and Brockhurst, 2012; Zwanzing et al., 2019; Baquero et al., 2021). Using nanopore long read sequencing, we identified most of the ARGs located on the chromosome and, surprisingly, only a few genes on plasmids (Figure 4A). *E. hormaechei* 120410-04 harbors a conjugative plasmid carrying resistance genes for colistin, aminoglycoside, trimethoprim, sulfonamide, quaternary ammonium, tetracycline, and beta-lactam. These genes are flanked by class 1 integrons, IS26, and Tn*3* family transposases. The plasmid is classified as incompatibility group IncHI2A, which is a well-known and widely distributed big plasmid in Enterobacter and known to carry multiple ARGs and BMRGs and was identified in other Enterobacterales, including *Salmonella, Escherichia*, and *Klebsiella* (Algarni et al., 2024). The vancomycin-resistance cassette from the *E. faecium* isolate was located on a conjugative plasmid. Similar plasmids harboring the *vanA* gene cluster in the Tn*1546* transposon were described before for multidrug-resistant *E. faecium* (Arredondo-Alonso et al., 2020). Plasmids are vehicles for horizontal gene transfer and enable fast transfer of ARGs to other bacteria, especially in environments with high antibiotic selection pressure (Harrison and Brockhurst, 2012). The copper resistance genes in proximity to the *vanA* gene cluster (Figure 4C) can further lead to co-selection through copper additives in feed, especially in pig farming. Furthermore, this *E. faecium* isolate carries chromosomal resistance genes for aminoglycosides, tetracycline, and macrolide/streptogramins. When antibiotic selection pressure in the environment is low, the chromosomal location of ARGs can benefit bacteria because resistance genes are inherited vertically, providing stability and ensuring the persistence of the genes. Furthermore, they profit from reduced fitness costs associated with plasmid maintenance and no risk of plasmid loss or instability (Zwanzing et al., 2019). In the food sector, the selective pressure of antibiotics may be lower than in clinical environments, thus providing an advantage for the chromosomally encoded ARGs. For instance, it has been demonstrated that foodstuff isolates possess the highest proportion of ESBL genes located on chromosomes, whereas clinical isolates exhibit a higher proportion on putative plasmids (García-Martín et al., 2024). Resistance genes located on the chromosome can be mobilized when associated with Integrative And Conjugative Elements (ICE), Transposons (Tn), integrons (Partridge et al., 2018). In *P. vulgaris* 121284-5, the aminoglycoside (*aadA1*), streptothricin (*sat2*), trimethoprim (*dfrA1*) resistance genes are located adjacent on a unit transposon (Tn*7*) (Figure 4C). This is a common resistance gene cassette array, a class 2 integron carrying *dfrA1-sat2-aadA1* located in a Tn*7* transposon (Mokracka et al., 2012; Siebor et al., 2018). These integrons facilitate the horizontal transfer of resistance traits among bacteria, contributing to the persistence of these genes in the environment and food chain (Ghaly et al., 2017). Although we found a few resistance genes on plasmids, ARGs were frequently located within or adjacent to mobile genetic elements, indicating their potential for mobilization to other bacterial hosts. However, further studies are needed to determine whether this enables the active transfer of ARGs to other bacteria, such as those in the human gut microbiome.

### 4.6 Status of antibiotic resistance in ready-to-eat meat products in Switzerland

This study presents the current status of AMR in RTE meat products in Switzerland. According to the surveillance programme, which focuses on ESBL/AmpC-producing *E. coli* and carbapenemase-producing *E. coli* and *Klebsiella spp.*, AMR in slaughter animals and meat in Switzerland has been declining in recent years (Federal Office of Public Health and Federal Food Safety and Veterinary Office, 2024). The proportion of ESBL/AmpC-producing *E. coli* has been declining strongly since 2014, with a prevalence of 4.2% in poultry meat, ≤ 1% in pork and beef meat, 6.2% in fattening pigs, and 32.7% in slaughter calves. This study only assessed multidrug resistance in *E. coli* from poultry and turkey meat, with data missing for other meat products. The highest resistance rates against sulfonamides and trimethoprim were detected in *E. coli* from slaughter calves, followed by resistance to ampicillin, trimethoprim, and chloramphenicol. According to the report, up to 2023, no carbapenemase-producing *E. coli* were detected.

Here, we did not find any carbapenemase-producing Enterobacterales, although two *Enterobacter hormaechei* and one *Proteus vulgaris* were phenotypically resistant to the carbapenem ertapenem. This phenotype, known as ertapenem-only-resistant, is common in Enterobacterales that are resistant to ertapenem but susceptible to other carbapenems, often due to a combination of different mechanisms, including production of AmpC or ESBL, outer membrane porin deficiency, or production of efflux pumps (Weston et al., 2024). The highly multidrug-resistant isolate *E. hormaechei* 120410-04 harbors 14 different ARGs. While the quinolone, phenicol/quinolone, fosfomycin, and cephalosporin resistance genes are located on the chromosome, the resistance genes for penicillins, colistin, tetracyclines, aminoglycosides, sulfonamides, and trimethoprim are located closely together on a plasmid. Similar MDR *E. hormaechei* isolates were identified in imported retail shrimp (Indugu et al., 2020), in clinics (Soliman et al., 2020; Yuan et al., 2023), and sprouts (Leighton et al., 2024). In Switzerland, *mcr* genes conferring resistance to colistin have already been detected in *E. coli* strains from broilers, cattle, and pigs (Federal Office of Public Health and Federal Food Safety and Veterinary Office, 2024). Our findings show that many other species beyond *E. coli*, which is typically used as an AMR indicator, can harbor resistance to critical antibiotics. This highlights the importance of surveilling not only *E. coli* as an indicator species but also monitoring AMR in environmental species that may act as a reservoir of AMR. As exemplified, it is important to not only set up more phenotypic surveillance systems but also to leverage the power of whole-genome sequencing and metagenomic sequencing methods for monitoring antibiotic resistance in the food chain (Federal Office of Public Health and Federal Food Safety and Veterinary Office, 2024). Genomic combined with phenotypic surveillance needs to be performed following the One Health approach, taking into account the interconnection between human, animal, and environmental health. Hence, food chains, especially animal-based foods, play an important role in the dissemination of AMR, and more research is needed to quantify this impact.

## 5 Conclusion

Our study shows that RTE meat products collected from Swiss butcheries could serve as reservoirs for ARB. Even though we only found a few bacterial isolates that were resistant to important antibiotics, the risk of transmission could still be relevant since these products are typically consumed raw. The scale of this impact needs to be elucidated in future studies. Diverse bacteria with different antibiotic resistance patterns were detected, highlighting the importance of extending surveillance to additional target microorganisms, for example, VRE. Furthermore, the application of long-read whole-genome sequencing provides valuable support for monitoring the potential spread of ARGs in the food chain.

## Data Availability

All code used for statistical analysis and graphing and the datasets are available from Zenodo under https://doi.org/10.5281/zenodo.15676386. The bacterial genome data (raw reads of the Illumina and Oxford Nanopore sequencing) is available from the BioProject PRJNA1309299.
