# Supplementary material for: Phenotypic and genotypic characterization of antibiotic-resistant bacteria from Swiss ready-to-eat meat products
Source: Front Microbiol. 2025 Sep 10;16:1649307. doi: 10.3389/fmicb.2025.1649307 (PMC12457351; doi:10.3389/fmicb.2025.1649307)
Supplement: Supplementary file 1 [file Data_Sheet_1.zip › Supplementary File 1.docx]

Spread of antibiotic resistance in pathogens from Swiss raw meat products

Supplementary Material

**Lisa Thoenen^1^, Jörg Hummerjohann^1^, Livia Schwendimann^2^, Elisabet Marti^1^ ***

^1^Food Microbial Systems, Agroscope, Schwarzenburgstrasse 161, 3003 Bern, Switzerland

^2^Swiss Quality Testing Services (SQTS), Lerzenstrasse 16, 8953 Dietikon, Switzerland

*** Correspondence:**Elisabet Marti
elisabet.martiserrano@agroscope.admin.ch

# Supplementary Data

**Table S1** | **Metadata all phenotyped bacterial isolates:** This table contains detailed information about the taxonomic assignment of the meat isolates and the isolation methods.

**Table S2** | **Sequencing statistics short-read:** This table contains the sequence type MLST for selected isolates and the sequencing statistics such as the contig count, N50, GC-content, read counts, assembly base count, genome size, contig lengths, coverage and read base counts of the assmeblies of all isolates sequenced by Illumina method.

**Table S3** | **Sequencing statistics long-read:** This table contains the sequencing statistics such as the contig counts, length of contigs, GC-content, N50 and the Ani taxonomy assignment for all isolates sequenced by the Oxford Nanopore method.

**Table S4** | **Predictions sources of genomic sequences:** This table presents the results from SourceFinder, which uses machine learning to identify the origin of chromosome, plasmid, and phage-derived sequences for each contig in the ONT data.

**Table S5** | **Correlation of ARGs and BMRGs:** This table reports the p-values for each correlation (Pearson product-moment correlation) for co-occurrence of ARGs and BMRGs.

# Supplementary Figures and Tables

## Supplementary Figures


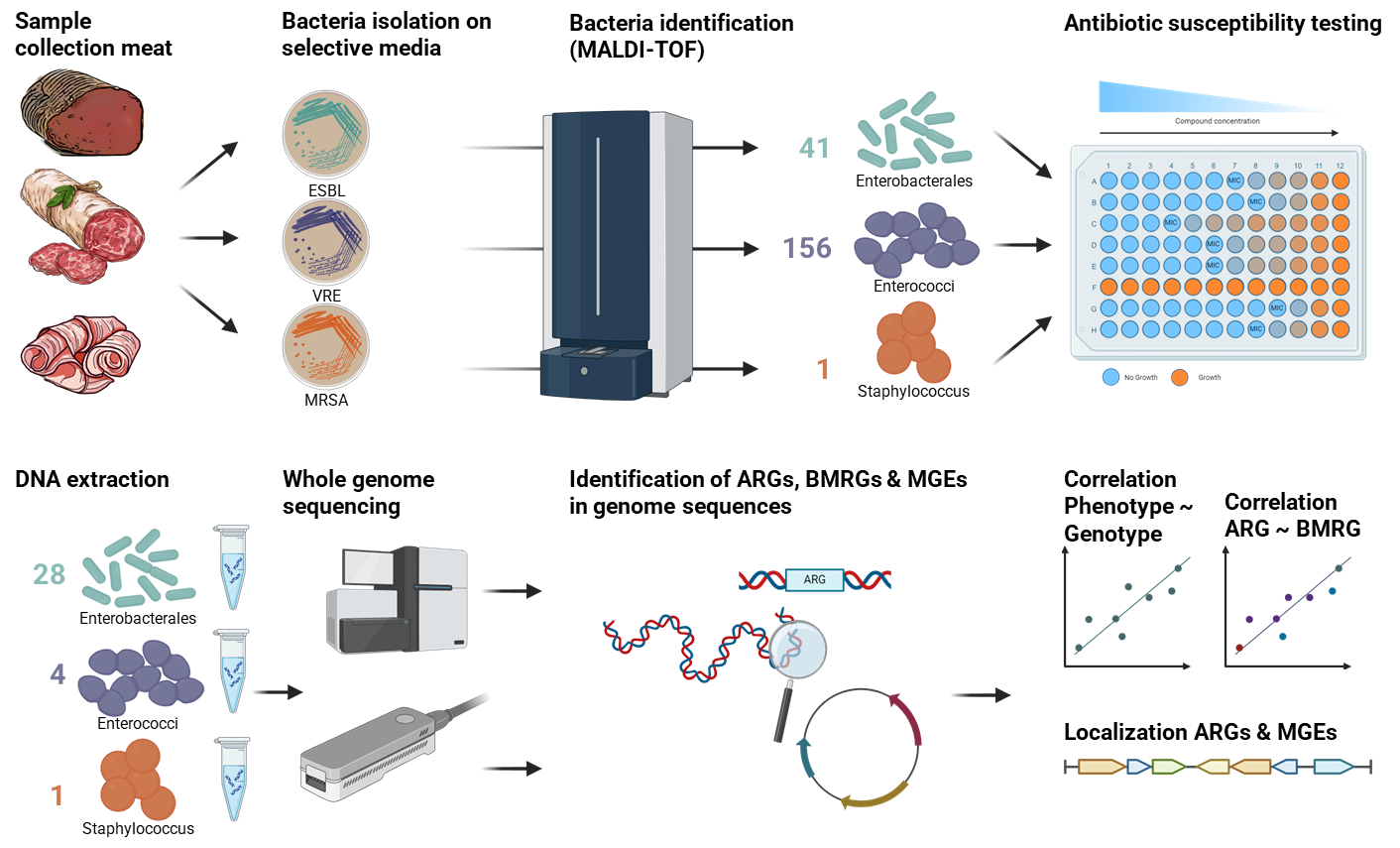
**Figure S1** | **Overview of study design and phenotypic and genotypic analysis performed.** Ready-to-eat meat products were collected in different Swiss butcheries. Presumptive antibiotic resistant bacteria were isolated on selective media for extended-spectrum beta-lactamase (ESBL) producing Enterobacterales, vancomycin resistant Enterococci (VRE) and methicillin resistant *Staphylococcus aureus* (MRSA). The taxonomy of the isolates was subsequently determined by Matrix Assisted Laser Desorption Ionization - Time of Flight (MALDI-TOF) and isolates identified as Enterobacterales, Enterococci and Staphylococcus were selected for further analysis. Antibiotic resistance phenotypes of the isolates were assessed using the semi-automated system based on the broth dilution method MicroScan. Isolates with interesting antibiotic resistance phenotypes were selected for short-read Illumina sequencing first. Based on the results of the first sequencing, several isolates were sequenced also by long-read Oxford Nanopore technique to get clearer results on the plasmid content of the bacterial isolates. In the sequencing results from both methods, antibiotic resistance genes (ARGs), biocide and metal resistance genes (BMRGs) and mobile genetic elements (MGEs) were identified using the bioinformatic tools AMRFinder and CGEMobile Element Finder. Further correlation analysis was conducted to examine the relationships between phenotypes and genotypes, as well as the presence of ARGs and BMRGs. Additionally, the localization of ARGs, BMRGs, and mobile genetic elements (MGEs) was analyzed.


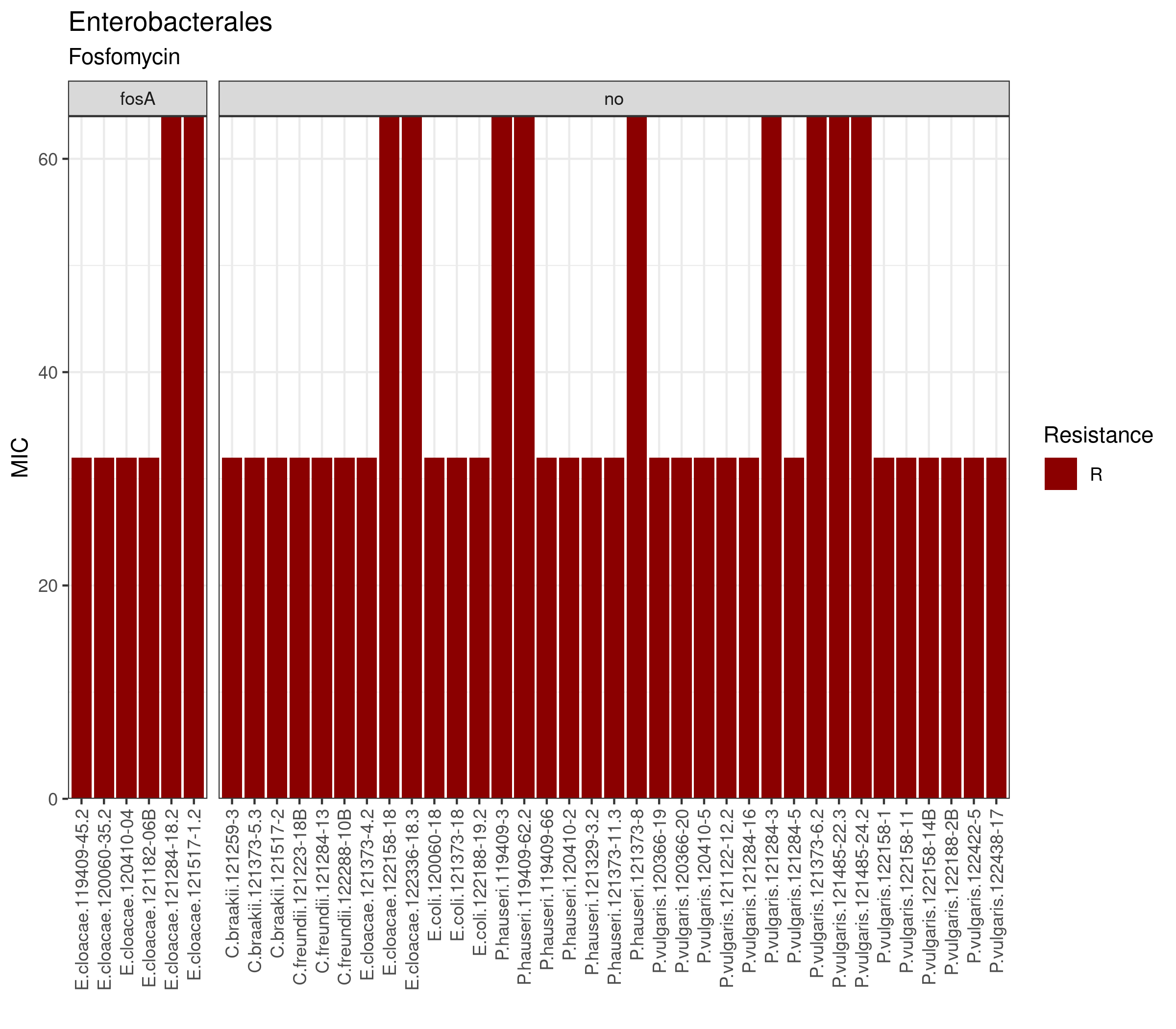
**Figure S2** | **Resistance of Enterobacterales to fosfomycin.** Bargraph showing the minimal inhibitory concentrations (MIC) of the tested Enterobacterales in fosfomycin. The isolates are grouped according to their genotype, carrying the fosfomycin resistance gene (fosA) or not (no). All isolates have a MIC above the threshold and thus considered resistant.


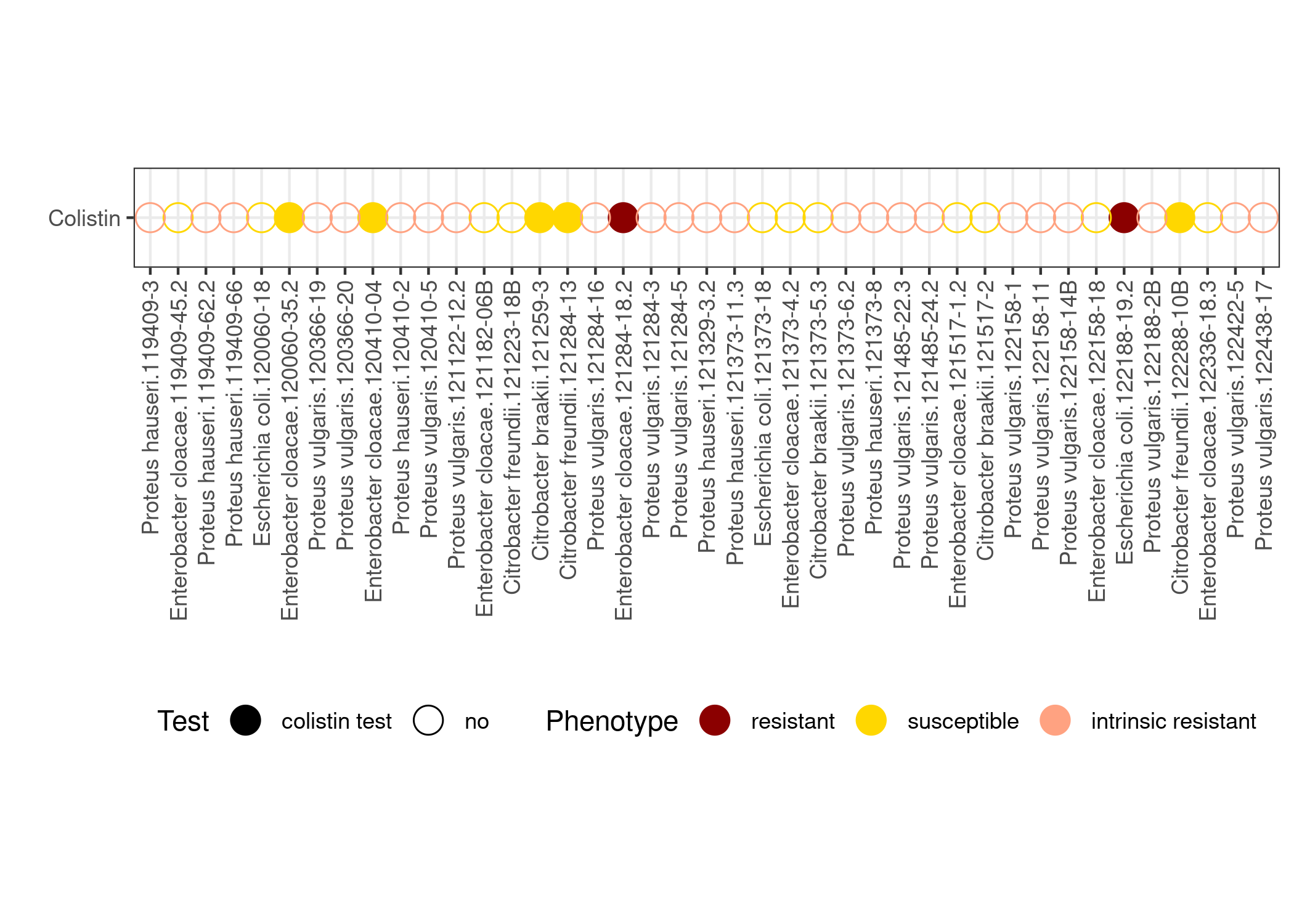


**Figure S3** | **Resistance of Enterobacterales to colistin.** Dotplot showing the results of the drop test to assess colistin resistance performed for the isolates with a filled circle. The color of the dots indicates the phenotype of each bacterial isolate, distinguishing them as resistant, intermediate, or susceptible.


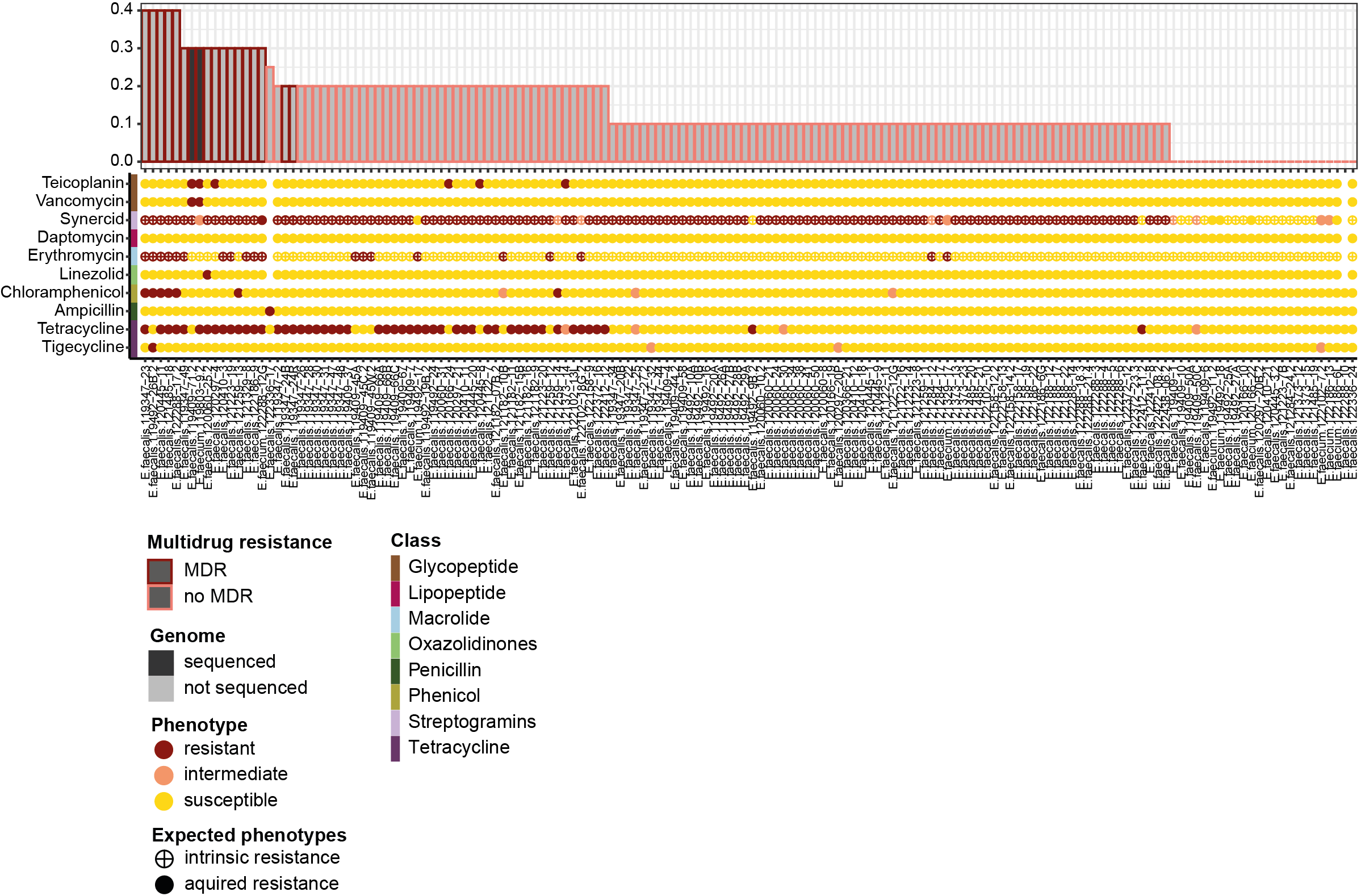


Figure S4 | Antimicrobial resistance phenotype of all tested Enterococci. Bargraph on top shows SMAR values of the bacterial isolates belonging to Enterococci (n = 156). Bars colored in black mark the isolates which were whole genome sequenced, while the colored borders indicate if they are MDR. Dot plot represents the phenotype of each bacterial isolate in the tested antibiotics classified as resistant, intermediate and susceptible.


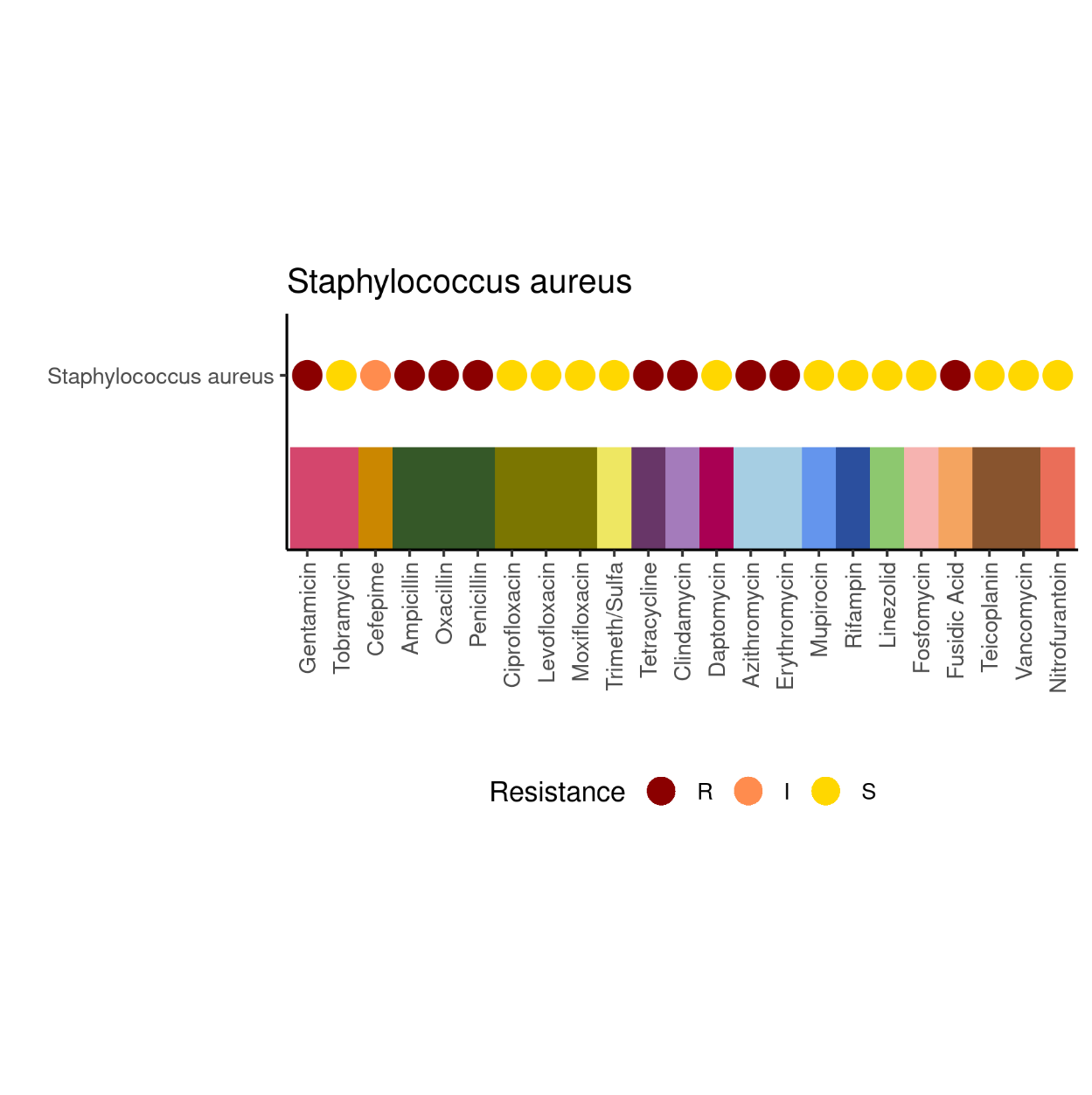

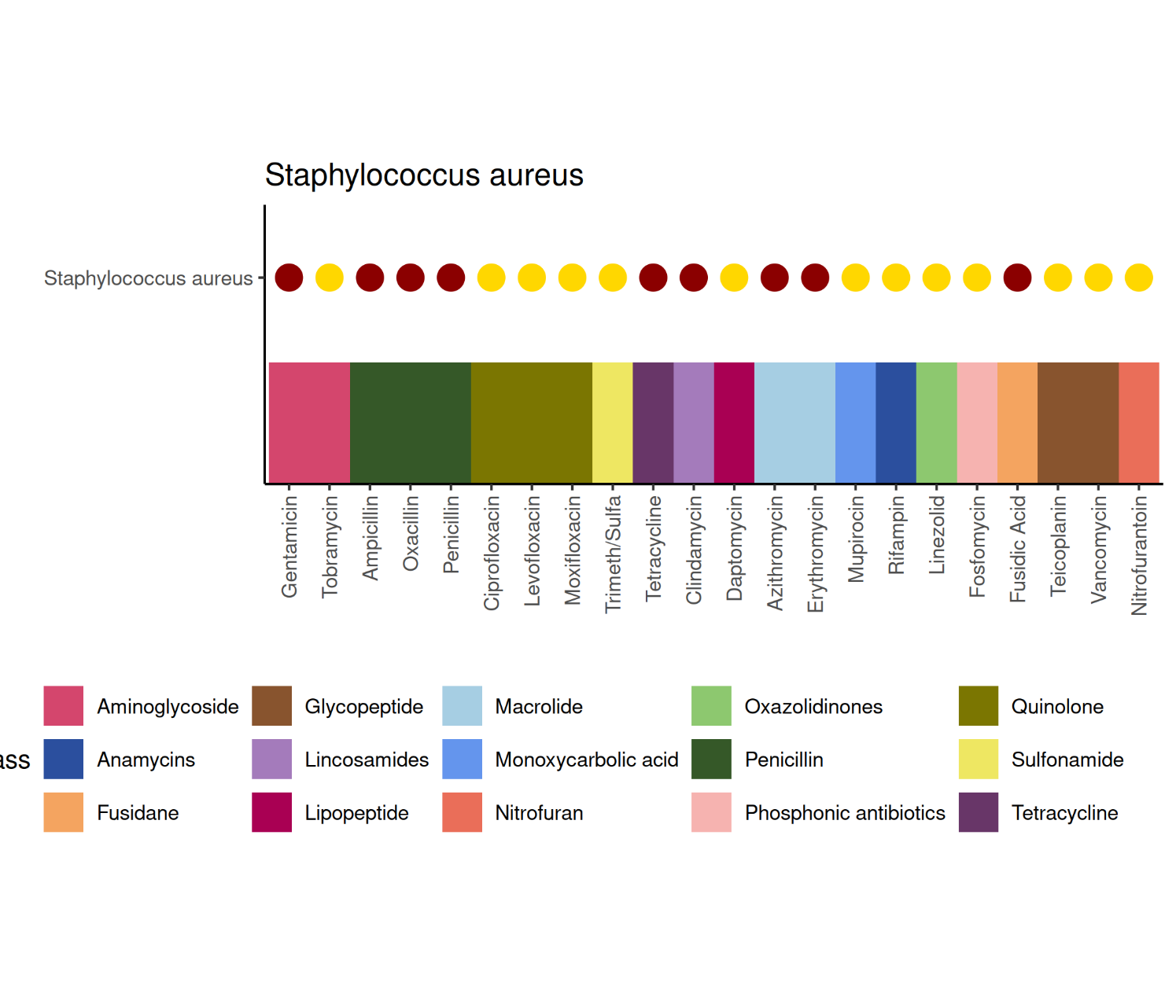


Figure S5 | Antimicrobial resistance phenotype of *Staphylococcus aureus* isolate. Dot plot represents the phenotype of each bacterial isolate in the tested antibiotics classified as resistant, intermediate and susceptible.


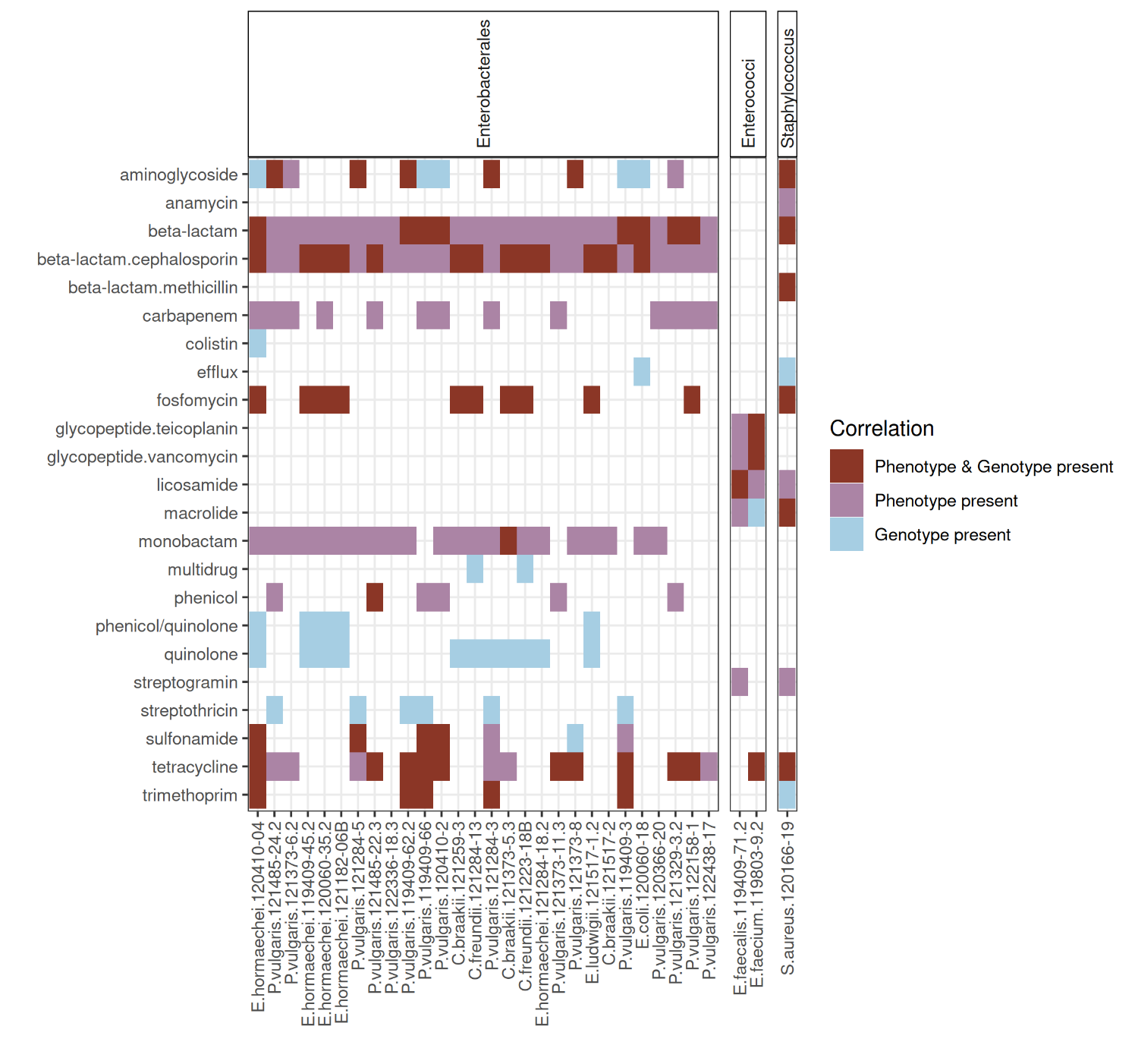


**Figure S6** | **Correlation antibiotic resistance phenotype and genotype grouped per antibiotic class.** This heatmap illustrates the correlation between antibiotic resistance phenotypes and genotypes, grouped by antibiotic class. Each cell represents the relationship for a specific antibiotic, with color coding based on the presence or absence of both phenotypes and genotypes.


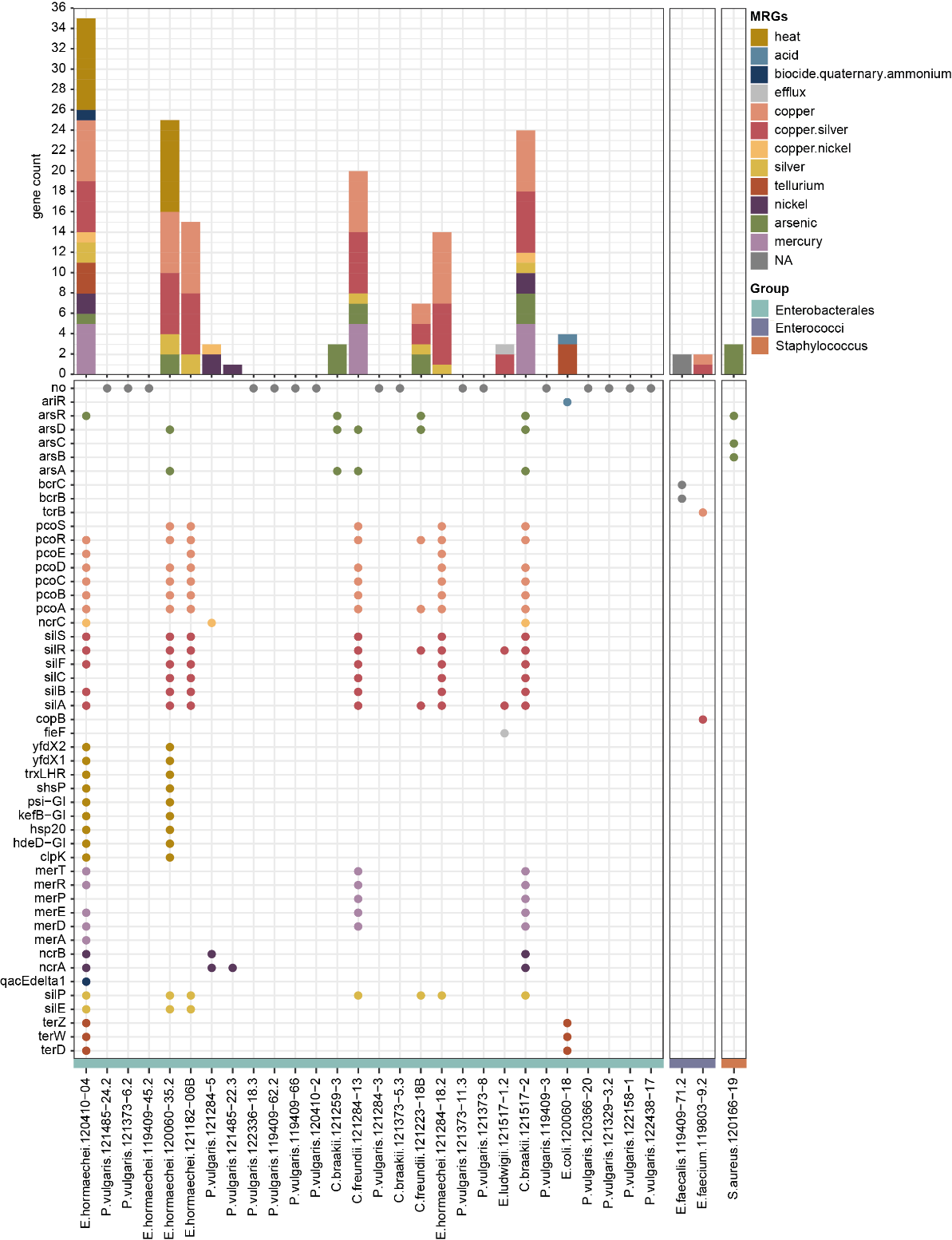


**Figure S7** | **Biocide and metal resistance genes in bacterial isolates from meat.** The BMRGs were identified using AMRFinder Plus in short read sequencing data. The upper bar graph displays the total number of BMRGs per strain, with colors representing different antibiotic classes. The lower dot plot presents individual genes (listed on the y-axis), where each dot is colored according to its corresponding antibiotic class.


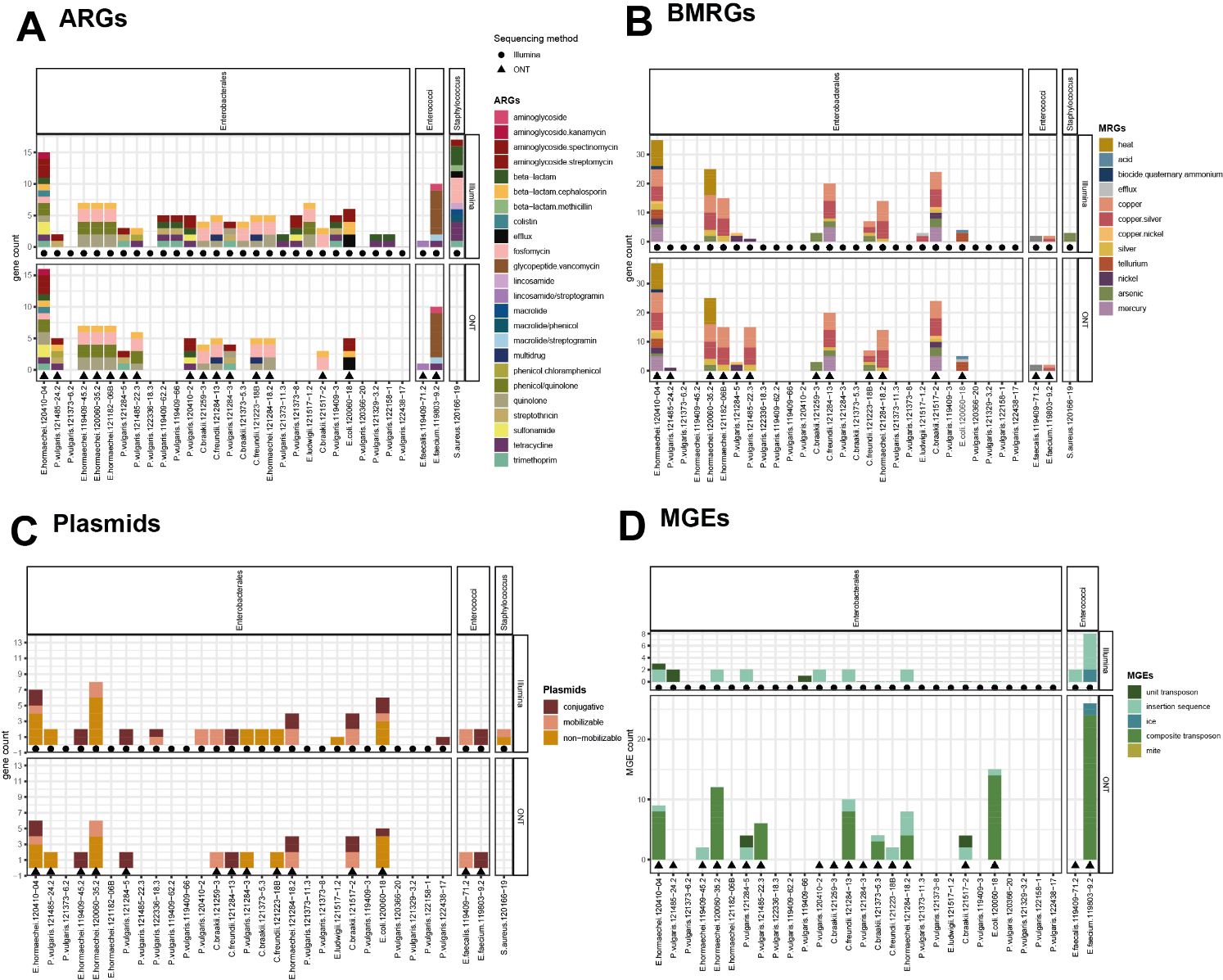


**Figure S8** | **Comparison of ARGs, BMRGs, plasmids and MGEs identified from short (Illumina) and long read (ONT) sequencing data.** Bargraphs display the total counts of the respective genetic elements colored according to the class of A) ARGs B) BMRGs C) plasmids and D) MGEs


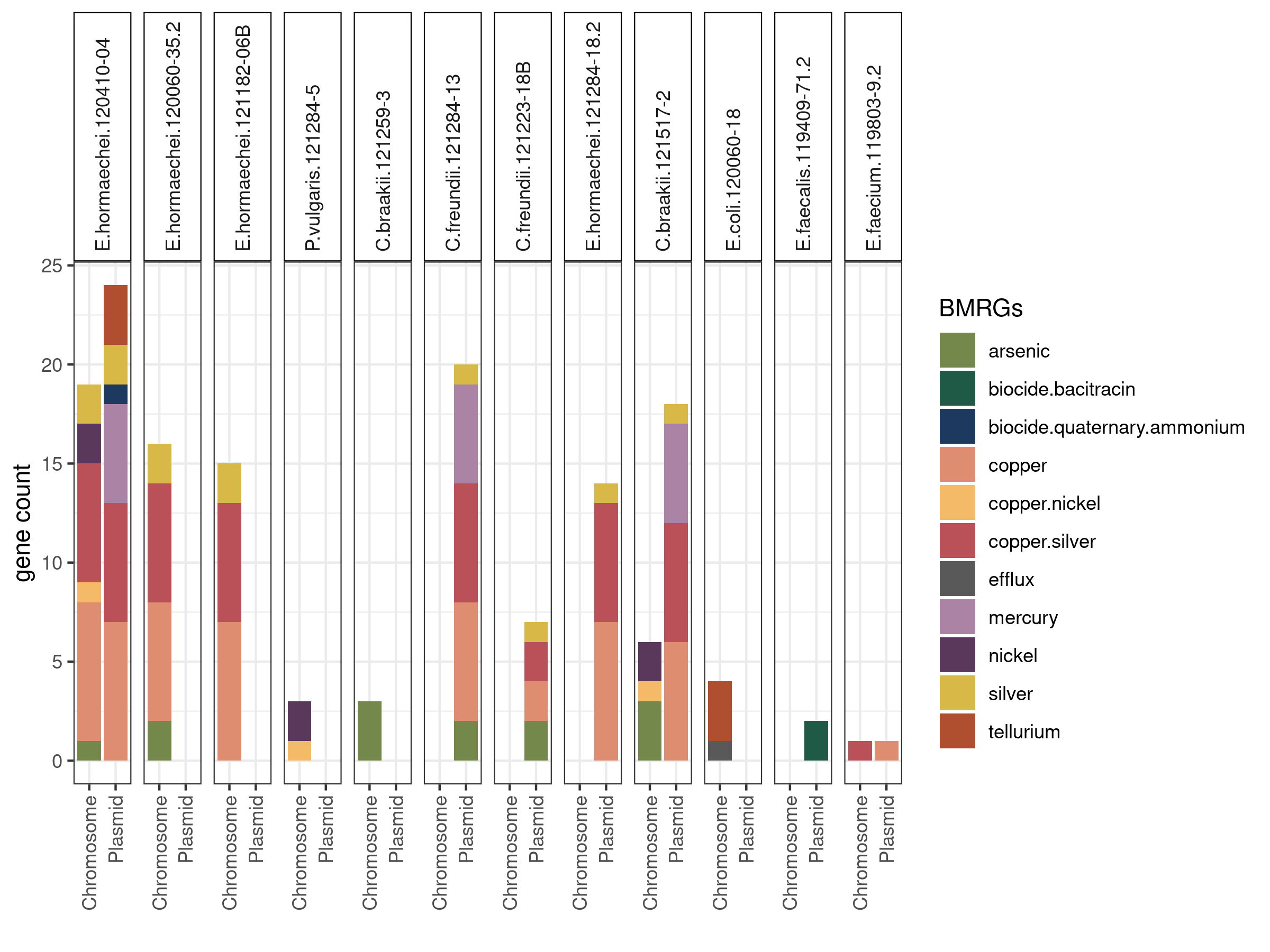


**Figure S9** | **Localization BMRGs on the chromosome or plasmid.** Long-read sequencing data were analyzed using SourceFinder and classified as chromosome or plasmid. Bargraph showing the count of BMRGs per strain and its location on the chromosome or plasmid. Bar is colored according to the class of BMRGs.
